# Unraveling the role of MAG, PTEN, and NOTCH1 in axonal regeneration: a network analysis and molecular dynamics study of siRNA/drugs/nanocarriers interactions

**DOI:** 10.1186/s12967-025-07042-9

**Published:** 2025-10-01

**Authors:** Alireza Salimi, Aysan Moeinafshar, Sima Rezvantalab, Mohammad Dabiri, Nima Rezaei, Nima Beheshtizadeh

**Affiliations:** 1 Roy J. and Lucille A. Carver College of Medicine, University of Iowa, Iowa City, IA 52242 USA; 2 School of medicine, Tehran University of Medical Sciences, Tehran, Iran; 3 Regenerative Medicine Group (REMED), Universal Scientific Education and Research Network (USERN), Tehran, Iran; 4 Chemical Engineering Department, Urmia University of Technology, 57166‑419, Urmia, Iran; 5 Department of Molecular Biology, Faculty of Natural Sciences, Comenius University in Bratislava, Ilkovičova 6, 841 04 Bratislava, Slovak Republic; 6 Department of Immunology, School of Medicine, Tehran University of Medical Sciences, Tehran, Iran; 7 Research Center for Immunodeficiencies, Children’s Medical Center, Tehran University of Medical Sciences, Tehran, Iran; 8 Network of Immunity in Infection, Malignancy and Autoimmunity (NIIMA), Universal Scientific Education and Research Network (USERN), Tehran, Iran; 9 Department of Tissue Engineering, Faculty of Advanced Medical Sciences, Tabriz University of Medical Sciences, Tabriz, Iran; 10 Stem Cell Research Center, Tabriz University of Medical Sciences, Tabriz, Iran; 11 Tabriz USERN Office, Universal Scientific Education and Research Network (USERN), Tabriz, Iran

**Keywords:** Drug delivery, siRNA delivery, Nervous system regeneration, Spinal cord injury recovery, Nanocarriers

## Abstract

**Background:**

Axonal regeneration remains a critical yet challenging process in spinal cord injury (SCI) recovery, primarily due to the limited regenerative capacity of adult central nervous system (CNS) axons. Identifying key molecular targets and optimizing therapeutic delivery systems are promising strategies to enhance axonal regeneration.

**Methods:**

In this study, we investigated the roles of three critical proteins—MAG, PTEN, and NOTCH1—in axonal regeneration through an integrative approach combining network analysis and molecular dynamics (MD) simulations. We compiled 361 regeneration-associated genes from the REGene database and a targeted PubMed literature review. Gene ontology enrichment analysis via DAVID identified key genes linked to axonal regeneration and oligodendrocyte differentiation. A protein-protein interaction (PPI) network was constructed to pinpoint hub genes, with Cytoscape used to assess degree, betweenness, and closeness centrality. The top-ranking genes across at least two centrality metrics were selected, and GeneMANIA validated their functional relevance, confirming MAG, PTEN, and NOTCH1 as negative regulators of regeneration. Using siDirect and siRNA Wizard, we designed siRNA molecules targeting these genes, while DGIdb and literature mining identified small-molecule drugs (e.g., GT1b for MAG, enzalutamide for PTEN). MD simulations explored their interactions with polymeric nanocarriers—PLGA, PEI, chitosan, and PEI-PEG—revealing distinct binding patterns.

**Results:**

All proteins exhibited favorable binding with their respective drugs, with MAG-GT1b demonstrating the strongest affinity ( −146.07 ± 61.63 kJ/mol). Free energy landscape (FEL) analysis of the MAG/GT1b complex revealed a pronounced global energy minimum at 20.6 kJ/mol, reflecting high-affinity binding. Among nanocarriers, chitosan showed strong siRNA interactions, whereas PLGA and PEI exhibited superior drug-binding properties, particularly for GT1b, as evidenced by lower solvent-accessible surface area (SASA) values, indicating tighter encapsulation. Notably, PLGA-based systems displayed a broader radius of gyration (Rg) distribution, attributed to their amphiphilic nature, which promotes rapid self-assembly into multiple dispersed nanocarriers rather than consolidated structures. Additionally, PLGA chains exhibited reduced average SASA values (40–90 nm^2^) compared to other polymers.

**Conclusions:**

The strongest siRNA interactions occurred between PTEN siRNA-enzalutamide and PLGA ( −107.31 kJ/mol) or PEI ( −87.15 kJ/mol), primarily driven by van der Waals forces. While these *in silico* findings are promising, preclinical validation is essential for clinical translation. This study highlights the potential of combining network analysis and MD simulations to decipher complex interactions among proteins, siRNA, drugs, and polymers, offering novel insights into therapeutic strategies for SCI.

**Supplementary Information:**

The online version contains supplementary material available at 10.1186/s12967-025-07042-9.

## Introduction

Central nervous system (CNS) injuries of different etiologies cause neuronal and axonal degeneration and subsequently, a severe neurological deficit. Despite their potential in dynamic differentiation and development, neurons possess a low regenerative capacity due to the inhibitory effects of their extracellular matrix (ECM) [1]. According to the 2019 Global Burden of Disease (GBD) study, spinal cord injury (SCI) is one of the most prevalent and common CNS injuries, with an incidence and prevalence of 0.9 and 20.6 million, respectively. The results of this study showed 6.2 million years-lived-with-disability (YLD) globally, indicating a significant disease burden [2].

The pathophysiology of SCI involves an acute phase, characterized by ischemia and subsequent vasogenic edema, as well as glutamate-mediated excitotoxicity; a subacute phase with mitochondrial phosphorylation and inflammatory processes; and finally, a chronic phase in which axonal degeneration and remodeling, demyelination, and scar formation occur [3]. Axonal regeneration is a cornerstone of recovery from SCI, and failure of this process is a primary cause of long-term functional disability.

Axonal regeneration can be promoted by inducing axonal sprouting and refining the injured neural circuits, as well as forming these circuits *de novo* to assist neurological and ultimately functional recovery [4]. The myelination of CNS neurons is performed by oligodendrocytes, which are differentiated from oligodendrocyte progenitor cells (OPCs) [5]. Due to altered distributions of ionic channels, SCI-associated oligodendrocyte cell death occurs acutely within minutes of injury and continuously within seven days, resulting in demyelination, and subsequently, axonal conduction block [6, 7]. OPC differentiation, maturation into oligodendrocytes, and subsequent myelin sheath formation are regulated via numerous signaling pathways and components, including the highly conserved pathways such as 1.1 *PI3K/Akt/mTOR*, *ERK/MAPK*, and *NOTCH* [8]

Several neurodevelopmental processes are mediated by the *NOTCH* pathway, including lateral inhibition, regulation of neurogenesis, dendrite remodeling, and differentiation of glial cells from their respective progenitor lines [9]. Unlike its role in other glial cell types, *NOTCH* pathway activation suppresses OPC differentiation into oligodendrocytes, primarily via *NOTCH1* receptor signaling [10].

Myelin-associated glycoprotein (MAG) is a transmembrane protein expressed by myelin-associated glia, Schwann cells, and oligodendrocytes [11]. This protein negatively regulates neurite outgrowth in regeneration, in contrast to its positive effects on nervous system growth. While *in vivo* studies suggest MAG deletion alone may not fully restore axonal regeneration, its potent inhibitory action *in vitro* and interaction with gangliosides like GT1b highlight its therapeutic potential as a local target [12, 13]. Furthermore, the phosphatase and tensin homolog (PTEN) is a tumor suppressor that exerts negative regulation of neuronal growth by inhibiting phosphatidyl inositol 3-kinase (*PI3K*), with its inhibition revealed to improve axon growth and promote oligodendrocyte survival in animal models of SCI [14, 15]. Genetic deficiency of this protein leads to defects in myelination due to oligodendrocyte dysfunction, abnormal synaptic transmission, and plasticity, and increased neurite density and caliber [16]. Finally, neurogenic locus notch homolog protein 1 (NOTCH1) is a component of the *NOTCH* signaling pathway.

In this study, we investigated negative regulators of neural regeneration in SCI, namely MAG, PTEN, and NOTCH1*,* via network analysis methods and specific regulatory RNAs. To this end, small molecule nanoplatforms have been designed *in silico* to induce spinal cord regeneration after injury. Utilizing network analysis methods, we can identify how the different negative regulators interact and regulate spinal cord regeneration. We further disrupted the functions of negative regulators using specific regulatory RNAs. This allows the nanoplatforms to more effectively induce spinal cord regeneration after injury. This strategy could ultimately lead to more effective treatments for SCI.

Nanoplatforms could also provide a platform for the delivery of therapeutic molecules, such as growth factors, to injured areas. Polymeric delivery systems such as poly(lactic-co-glycolic acid) (PLGA), Polyethylenimine (PEI), chitosan, and PEI- Polyethylene glycol (PEG) are among the effective candidates in this field and are used due to advantages such as biocompatibility, controlled release of the delivered agents, and low toxicity [17, 18].

Hence, this work aims to test the concept that inhibition of MAG, PTEN, and NOTCH1 using siRNAs and small molecule drugs, delivered by polymeric nanocarriers, may support axonal regeneration in the injured spinal cord. To test this hypothesis, we applied a systems biology approach combining gene ontology and network analyses to identify therapeutic targets, followed by molecular dynamics (MD) simulations to evaluate drug and siRNA interaction profiles with different polymers.

To unravel the biological functions of macromolecules, understanding their molecular interactions and structures is critical. MD simulations are a method of conducting such modeling and can be used to study the interactions of macromolecules such as proteins and nucleic acids with a variety of substances and their behavior within specific ensembles [19]. In this paper, MD simulations were used to evaluate the loading of siRNAs and drugs into polymers and their interactions with PLGA, PEI, chitosan, and PEI-PEG polymer strands.

## Materials and methods

### Genes ontology analyses

A systems biology investigation was conducted using the Regeneration Gene Database (REGene) [20] and a structured literature review using PubMed (keywords: “axonal regeneration”, “oligodendrocyte differentiation”, “SCI regeneration”, and “negative regulation”) to identify 361 regeneration-related genes (Tables S1 and S2). Given that the Regeneration Gene Database contains genes involved in the regeneration of human tissues and/or organs, all procedures were carried out in conformity with the applicable guidelines and regulations.

As part of the analysis of each gene obtained using the Database for Annotation, Visualization, and Integrated Discovery (DAVID), individual gene ontology (GO) terms were enriched to determine whether co-transcriptionally regulated genes are involved in axonal regeneration and oligodendrocyte differentiation. After entering our gene list into this database, 25 genes involved in axonal regeneration and 26 genes associated with oligodendrocyte differentiation, based on GO term enrichment, were selected for further analysis on these genes (Tables S3 and S4).

DAVID distinguishes itself from related databases by providing a built-in and enlarged back-end annotation database, advanced modular enrichment techniques, and robust exploratory functionality in a built-in data-mining context [21]. One of the most fundamental factors that directly influences the overall performance of DAVID’s functional analysis is the size of large gene lists obtained from high-throughput biological investigations [22].

The next tool we utilized to examine the protein‐protein interaction (PPI) arrangements of genes is the search tool for the retrieval of interacting genes, STRING v10.5, which is a web database, providing a platform for analyzing molecular interactions of genes or disease mechanisms through functional association networks of target gene products [23]. In this study, the STRING database was utilized to construct the PPI network of two gene lists associated with axonal regeneration and oligodendrocyte differentiation. To balance specificity and sensitivity, capturing less evident but potentially significant interactions, the combined score threshold of 0.4 in STRING was chosen. In related network studies, this threshold is frequently applied to minimize noise and preserve biologically significant interactions [24].

### Hub genes selection and analyses

Cytoscape v3.7.0 is an open‐access bioinformatics software that is utilized to draw and examine functional interaction networks of target genes downloaded from the STRING database [25]. The parameters that were applied were as follows: degree cut‐off = 2, node k‐score = 2, score cut‐off = 0.2, and max depth = 100 to focus on nodes that were not isolated or sparsely connected, improving the biological interpretability of network hubs.

Using Cytoscape, we identified hub genes based on degree, betweenness, and closeness centrality. Genes that ranked among the top in at least two of these three metrics, across two separate networks, were selected as hub genes. Degree centrality, defined as the number of connections per node in Cytoscape, represents a gene’s connectivity within the network (Tables S5 and S6).

Accordingly, six hub genes from two separate networks were entered into the GeneMANIA database to identify potential partners for negative regulation of regeneration [26]. In this database, genes involved in negative regulation of regeneration were determined by selecting functions: negative regulation of growth, negative regulation of neurogenesis, negative regulation of cell development, and negative regulation of nervous system development. Then the Enrichr database was used to confirm the results of GeneMANIA [27]. Targeting genes involved in the negative regulation of regeneration with specific inhibitors could promote CNS repair, particularly after SCI. Aiming at this concept, finding the optimal siRNAs and small molecules was our next step.

For siRNA design, the mRNA sequences of the target genes were extracted from the NCBI Nucleotide database [27]. The sequences were used in two different siRNA prediction tools: siDirect [28] and siRNA Wizard [29], and the siRNAs described in both results were primarily chosen. If there were no overlaps among the results of the two prediction tools, all results were added to the primary list of candidates. To assess off-target effects and ensure target specificity, each siRNA candidate was analyzed using NCBI BLAST against the human RefSeq mRNA database. Only those siRNAs that aligned uniquely to the intended gene target with a perfect match and showed minimal or no significant off-target alignment, particularly within the seed region (nucleotides 2–8), were selected for further study. In addition, secondary structure predictions were conducted using RNAfold (ViennaRNA WebSuite) [30]. Candidates with minimal internal base pairing (MFE close to 0 kcal/mol) and high ensemble frequency of unstructured conformations (>90%) were prioritized. RNAComposer was employed for RNA structure modeling in MD simulations [31, 32]. In this study, we used the interactive mode of RNAComposer. In this regard, we entered three siRNA sequences separately.

Hence, siRNA design was conducted according to the methods, and the candidates were chosen by the highest compatibility with the target sequences in the genetic loci, as well as the least off-target interaction possible. The resulting RNA sequences and their target positions in the RNA interference processes have been summarized in Table 1.

**Table 1:** The siRNA candidates designed to target MAG, PTEN, and NOTCH1

	MAG	PTEN	NOTCH1
siRNA sequence (guide)	UUGAAGAUGGUGAGAAUAGGG	AACUUUGUAUUCACAUUUGGC	UCUUGUAGGGGAUGUUGAGGC
siRNA sequence (passenger)	CUAUUCUCACCAUCUUCAAGG	CAAAUGUGAAUACAAAGUUUU	CUCAACAUCCCCUACAAGAUC
Target sequence	CCCTATTCTCACCATCTTCAAGG	GCCAAATGTGAATACAAAGTTTT	GCCTCAACATCCCCTACAAGATC
Position	1185-1207	5436-5458	5394-5416

### Drug analysis

GT1b was selected due to its role as a competitive binding partner that disrupts MAG-mediated inhibition of neurite outgrowth [33]. Enzalutamide, an androgen receptor inhibitor, has been reported to indirectly modulate PTEN signaling and was identified through DGIdb as a potential modulator [34]. We chose the γ-secretase inhibitor Crenigacestat because it has been shown to suppress *NOTCH1* signaling by blocking the cleavage and activation of the *NOTCH* intracellular domain [35]. We analyzed drug-siRNA interactions for NOTCH1 and PTEN (identified via databases) and MAG (identified via literature review [36]). The analysis could determine which drugs can inhibit the targeted genes.

### MD simulations

**Fig. 1:**
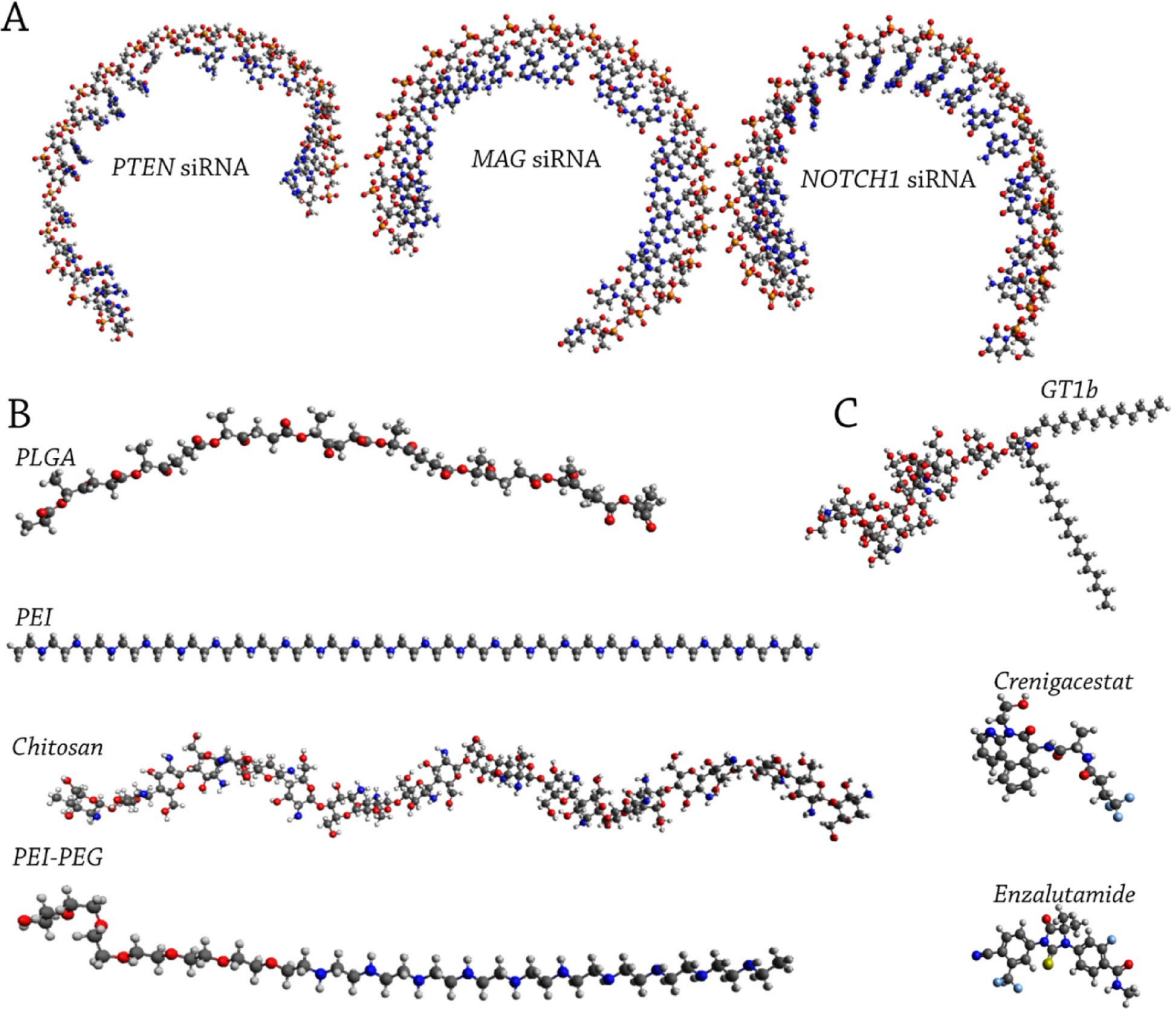
Snapshots of MD simulation inputs: **A** siRNAs, **B** Polymers, and **C** Drugs

All-atom MD (AA-MD) simulations were performed to study siRNA-drug-protein interactions. PLGA, PEI, chitosan, and PEI-PEG polymers used in the study were linear and prepared using the CHARMM-GUI polymer builder [37]. Figure 1 shows the molecular structures of the three siRNAs, drugs, and polymers used as simulation inputs. Drug and polymer topologies were generated using the PolyParGen web server [38]. The topologies of the siRNAs were generated using the pdb2gmx command in GROMACS 2020.7 software.

Multiple simulations were designed and executed, as shown in Table 2. Components were solvated in cubic boxes (90 Å × 90 Å × 90 Å) with SPC/E water and 20 randomly placed Na⁺ ions. Na^+^ ions were added to neutralize system charge. Each simulation included 10 polymer chains interacting with siRNA, drugs, or both simultaneously. All simulations were performed with the AMBER99 force field.

**Table 2:** Molecular compositions of MD simulations

NO.	siRNA	Drug	Polymer
1	PTEN	–	PLGA
2	–	Enzalutamide	PLGA
3	PTEN	Enzalutamide	PLGA
4	MAG	–	PLGA
5	–	GT1b	PLGA
6	MAG	GT1b	PLGA
7	NOTCH1	–	PLGA
8	–	Crenigacestat	PLGA
9	NOTCH1	Crenigacestat	PLGA
10	PTEN	–	PEI
11	–	Enzalutamide	PEI
12	PTEN	Enzalutamide	PEI
13	MAG	–	PEI
14	–	GT1b	PEI
15	MAG	GT1b	PEI
16	NOTCH1	–	PEI
17	–	Crenigacestat	PEI
18	NOTCH1	Crenigacestat	PEI
19	PTEN	–	Chitosan
20	–	Enzalutamide	Chitosan
21	PTEN	Enzalutamide	Chitosan
22	MAG	–	Chitosan
23	–	GT1b	Chitosan
24	MAG	GT1b	Chitosan
25	NOTCH1	–	Chitosan
26	–	Crenigacestat	Chitosan
27	NOTCH1	Crenigacestat	Chitosan
28	PTEN	–	PEI-PEG
29	–	Enzalutamide	PEI-PEG
30	PTEN	Enzalutamide	PEI-PEG
31	MAG	–	PEI-PEG
32	–	GT1b	PEI-PEG
33	MAG	GT1b	PEI-PEG
34	NOTCH1	–	PEI-PEG
35	–	Crenigacestat	PEI-PEG
36	NOTCH1	Crenigacestat	PEI-PEG

The second phase of MD simulations examined protein-drug interactions. Protein structures were retrieved from the Protein Data Bank (PDB). Table 3 lists protein PDB codes and their associated drugs. We removed ligands from protein structures using ViewerLite [39]. Missing loops were repaired via energy minimization steps in Swiss-PDB Viewer. Proteins were solvated in cubic boxes (90 Å × 90 Å × 90 Å). The system was neutralized with Na⁺ ions and solvated with SPC/E water.

**Table 3:** Protein PDB IDs and associated drugs used in MD simulations

NO	Protein	PDB code	Drug
1	MAG	5flr	GT1b
2	PTEN	1d5r	Enzalutamide
3	NOTCH1	3l95	Crenigacestat

Simulations used periodic boundary conditions over 100 ns. Hydrogen bonds (H-bonds) were constrained with the LINCS algorithm [40] using 2 ps time steps. Electrostatic interactions were calculated via the Particle Mesh Ewald (PME) method [41]. Pressure coupling was maintained with the isotropic Parrinello-Rahman algorithm [42]. Systems were maintained at 1 bar (Berendsen barostat) and 300 K (Berendsen thermostat), with a 1.0 nm Coulomb cutoff and 1.4 nm van der Waals (vdW) cutoff. Trajectories were visualized using Visual Molecular Dynamics (VMD) [43].

### Solvent-accessible surface area (SASA)

To assess protein surface interactions, we calculated the solvent-accessible surface area (SASA), which quantifies the biomolecular surface exposed to the solvent. This metric provides insights into molecular packing and aggregation states; a reduction in SASA indicates increased protein-protein or protein-ligand interactions, reflecting higher aggregation or binding density.

### Binding free energy analysis

For binding energetics, we employed the Molecular Mechanics Poisson-Boltzmann Surface Area (MM-PBSA) method [44], which evaluates electrostatic, vdW, and solvation contributions to intermolecular interactions. To enhance computational accuracy, we utilized g_mmpbsa, an open-source tool, addressing limitations of traditional MM-PBSA implementations. The total binding free energy (ΔG_bind_) was decomposed into:

- Molecular mechanics contributions (ΔG_MM_): Electrostatic and vdW interactions.
- Solvation contributions (ΔG_solv_): Polar terms derived from Poisson-Boltzmann calculations (dielectric constants: ε_protein_ = 2, ε_water_ = 80) and nonpolar terms estimated from SASA (γ = 0.00542 kcal/mol/Å^2^).

### Free energy landscape (FEL) analysis

The FEL analysis provides a thermodynamic perspective on protein conformational stability by mapping accessible states in terms of Gibbs free energy [45]. This approach identifies low-energy conformations and transition states, offering insights into the dynamic behavior of protein-drug complexes [46]. In this study, the first two principal components (PC1 and PC2), derived from MD trajectories, were used to construct the FEL for each simulated protein-ligand system. Gibbs free energy calculations were performed using the g_sham tool implemented in GROMACS 2020.7, enabling the visualization of energetically favorable conformations and potential intermediate states.

### Dynamic cross-correlation matrix (DCCM) analysis

To investigate residue-level protein dynamics, the cross-correlation matrix of C-α atomic displacements was computed from MD simulations. This analysis revealed both correlated and anti-correlated motions within the protein structure, highlighting regions that move in concert or opposition during interaction with the ligand [47]. Principal component analysis (PCA) was employed to reduce the dimensionality of the covariance matrix, where the eigenvalues and eigenvectors were derived to quantify dominant modes of atomic fluctuations. These modes capture the most significant collective motions governing protein flexibility and ligand-induced conformational changes [48]. All simulations and subsequent analyses were conducted using GROMACS 2020.7, ensuring consistency in computational methodology.

## Results

### Gene ontology analyses

**Fig. 2:**
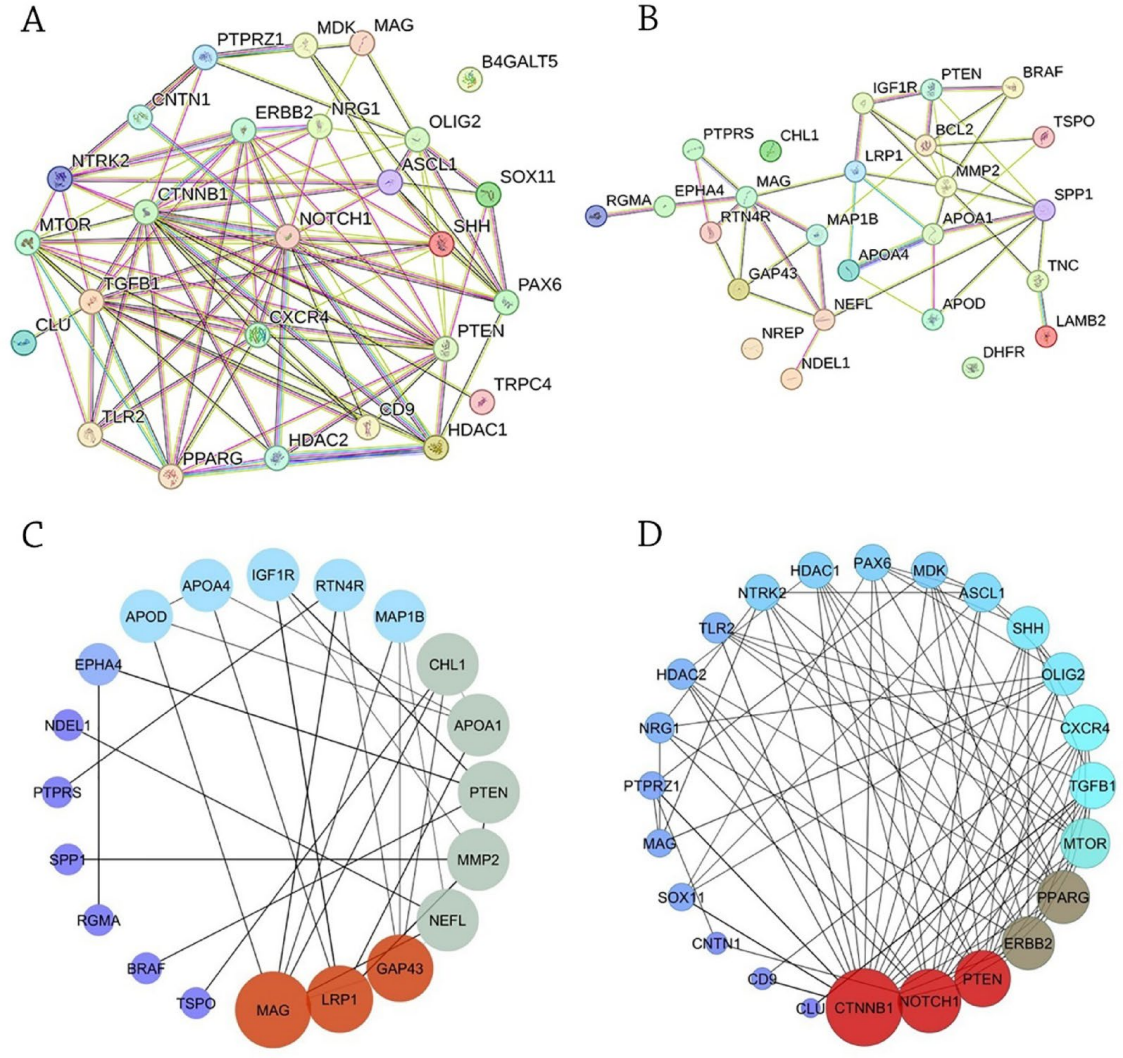
Protein-protein interaction networks. **A** Oligodendrocyte differentiation-associated network. **B** Axonal regeneration-associated network. Both networks were generated using the STRING Database. **C** Hub genes in axonal regeneration network. *MAG*, *GAP43*, and *LRP *show highest degree centrality (red nodes). **D** Hub genes in oligodendrocyte differentiation network. *CTNB*, *NOTCH1*, and *PTEN* show highest degree centrality (red nodes). Node color intensity (red to blue) represents degree centrality (high to low).

Using the DAVID database, we evaluated 361 genes (285 from the regeneration gene database [20] and 76 protein-coding genes from the literature, Tables S1 and S2). The results identified 25 genes that play significant roles in axonal regeneration. Additionally, 26 genes have been identified as being important in the differentiation process of oligodendrocytes. Oligodendrocytes are specialized cells that produce myelin, a fatty substance that insulates axons and facilitates their conduction of electrical signals. Figure 2A shows a network of genes associated with oligodendrocyte differentiation.

Axonal regeneration is a complex process that involves the activation of various signaling pathways and the expression of specific genes. The STRING database provides a useful tool to explore the PPI underlying this process using genes gathered from the DAVID database. Figure 2B shows a network of genes associated with axonal regeneration.

**Fig .3:**
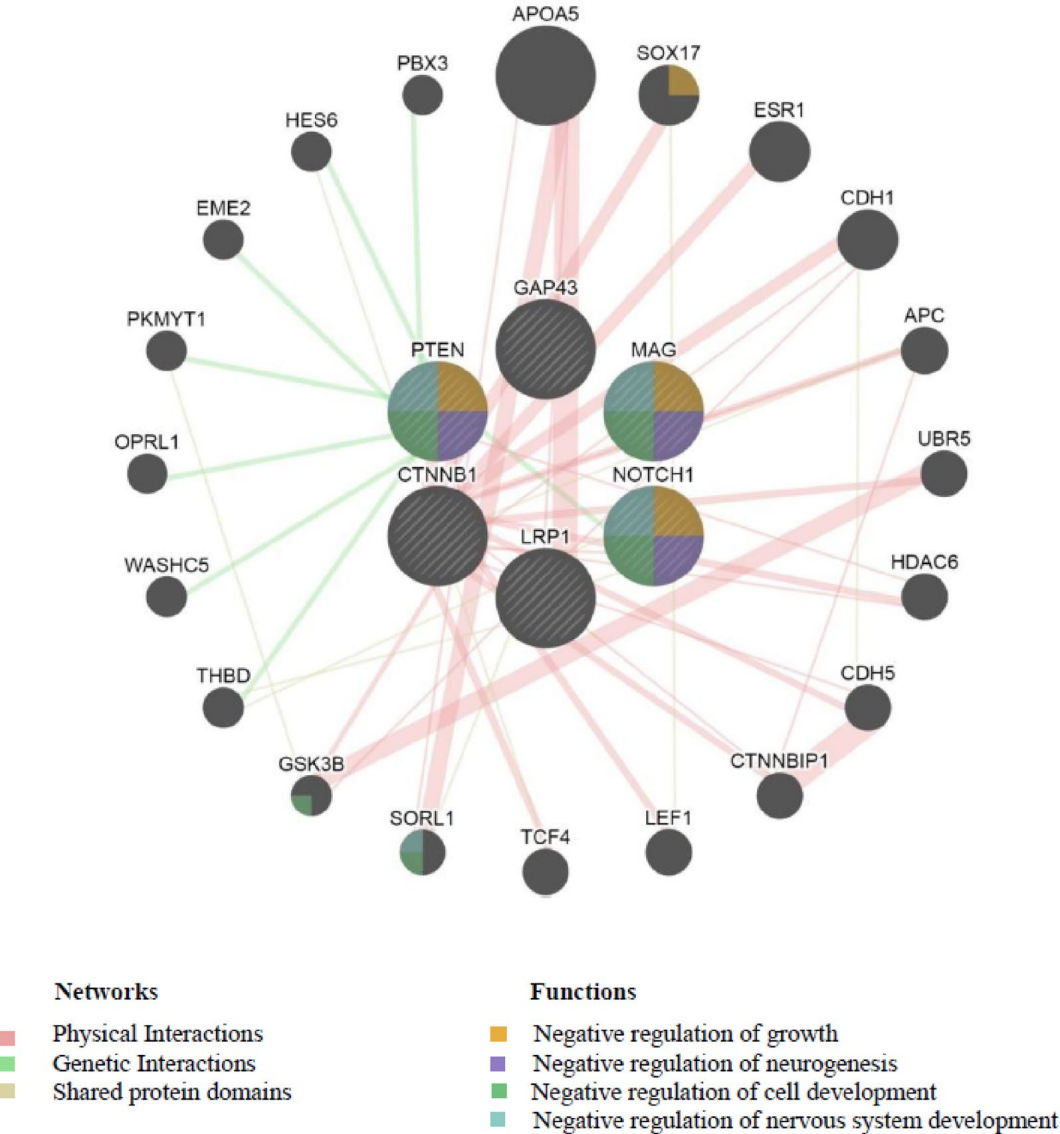
Network resulting from GeneMANIA Database analysis. *MAG*, *NOTCH1*, and *PTEN* were identified as key regulators of selected functions involved in nerve regeneration.

Hub gene analysis in Cytoscape identified *MAG*, *GAP43*, and *LRP1* as top-ranked genes in the axonal regeneration network based on their highest degree centrality (Fig. 2C). In addition, oligodendrocyte differentiation-associated network hub gene analysis revealed *CTNB*, *NOTCH1*, and *PTEN* as potential genes with degrees of 19, 15, and 13, respectively (Fig. 2D). To further analyze these genes, we entered them into the GeneMANIA database (Fig. 3). In this database, we found MAG, NOTCH1, and PTEN as significantly associated with selected functions that play critical roles in nerve regeneration: negative regulation of growth, negative regulation of neurogenesis, negative regulation of cell development, and negative regulation of nervous system development. The Enrichr database confirmed the significant association of MAG, NOTCH1, and PTEN with the negative regulation of axonogenesis and the negative regulation of neurogenesis.

### MD simulations

#### Protein-drug interactions

**Fig. 4:**
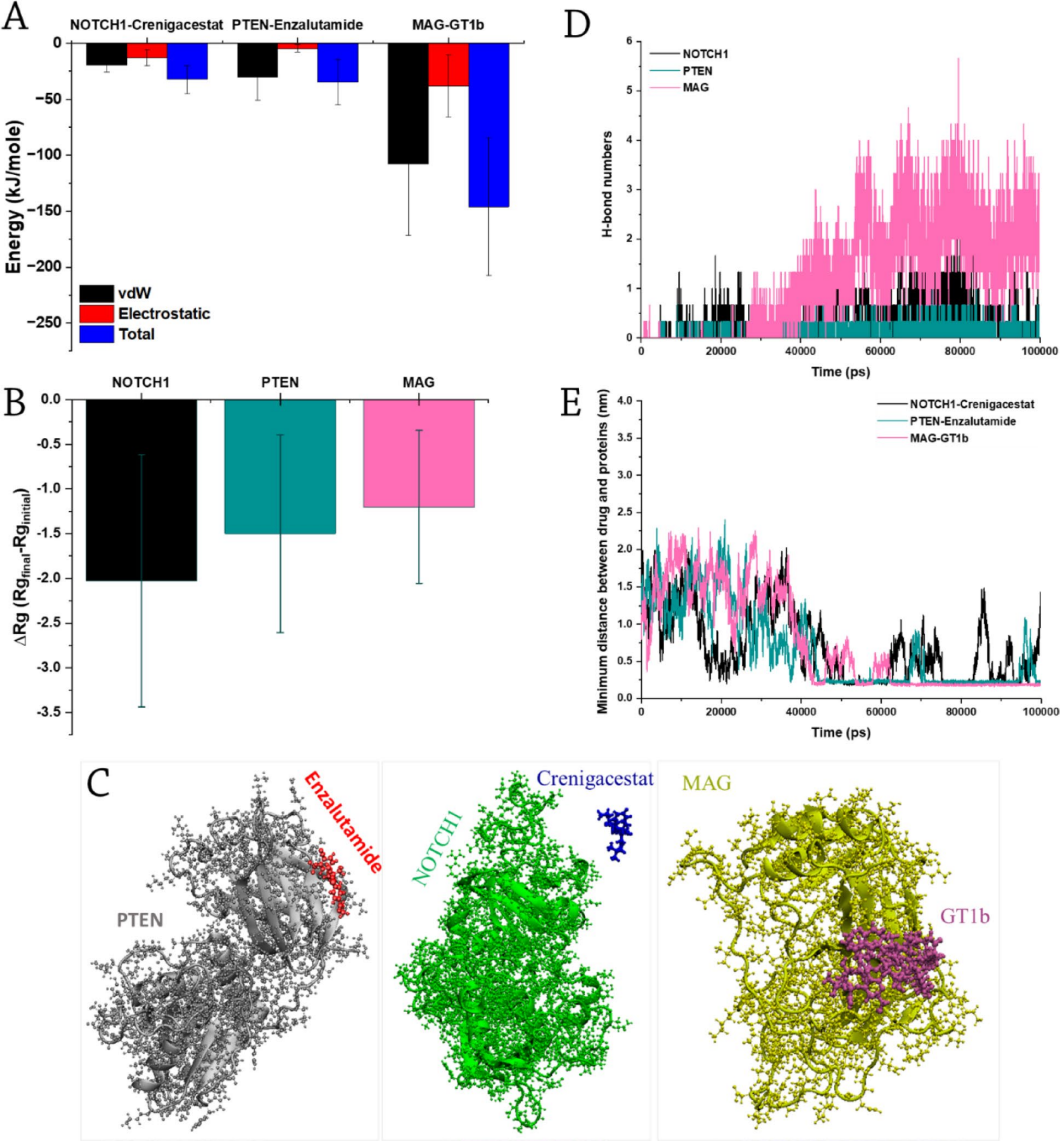
**A** Average protein-drug interaction energies (n=3); **B** ΔR_g_ (final-initial, n=3); **C** Final simulation snapshots; **D** Average H-bond counts; **E** Minimum distances between proteins and drugs at the simulation time. (vdW=Van der Waals).

To ensure robustness, each parameter set was evaluated over three independent simulation runs. Average energy components (reflecting interaction magnitude) were calculated per simulation (Fig. 4A; full data in Figures S1A, B and C). All proteins showed attraction to their corresponding drugs (negative energy values), with the strongest interaction observed between MAG and ganglioside GT1b ( −146.07 ± 61.63 kJ/mol). vdW interactions dominated in all cases (e.g., −107.88 kJ/mol, 74% of total energy for MAG-GT1b). PTEN-enzalutamide showed the highest vdW contribution (87% of total energy). Energy changes >20 kJ/mol are biologically significant in protein-ligand interactions, as they correlate with 10- to 100-fold changes in binding constant (K_d_) [49]. Stronger vdW interactions may thus enhance binding/delivery efficiency.

Radius of gyration (Rg) trajectories are shown in Figure S1D. We report the average ΔRg (final-initial Rg difference across replicates) to quantify drug-induced volume changes (Fig. 4B). Positive ΔRg indicates expansion (drug binding), while negative values suggest compaction. Stronger interactions (e.g., MAG-GT1b) correlated with smaller ΔRg (∼1 nm decrease). Simulation snapshots are shown in Fig. 4C.

H-bond analysis (Figures S1E and 4D) revealed increasing H-bonds for MAG-GT1b (strong interaction), while PTEN-enzalutamide maintained only sporadic H-bonds (mean=1), indicating weak affinity. Minimum protein-drug distances (Fig. 4E and S1F) showed stable MAG-GT1b binding (zero distance after 40 ns), whereas PTEN-enzalutamide dissociated transiently before rebinding. NOTCH1-crenigacestat remained separated.

#### Dynamic and thermodynamic analyses

**Fig. 5:**
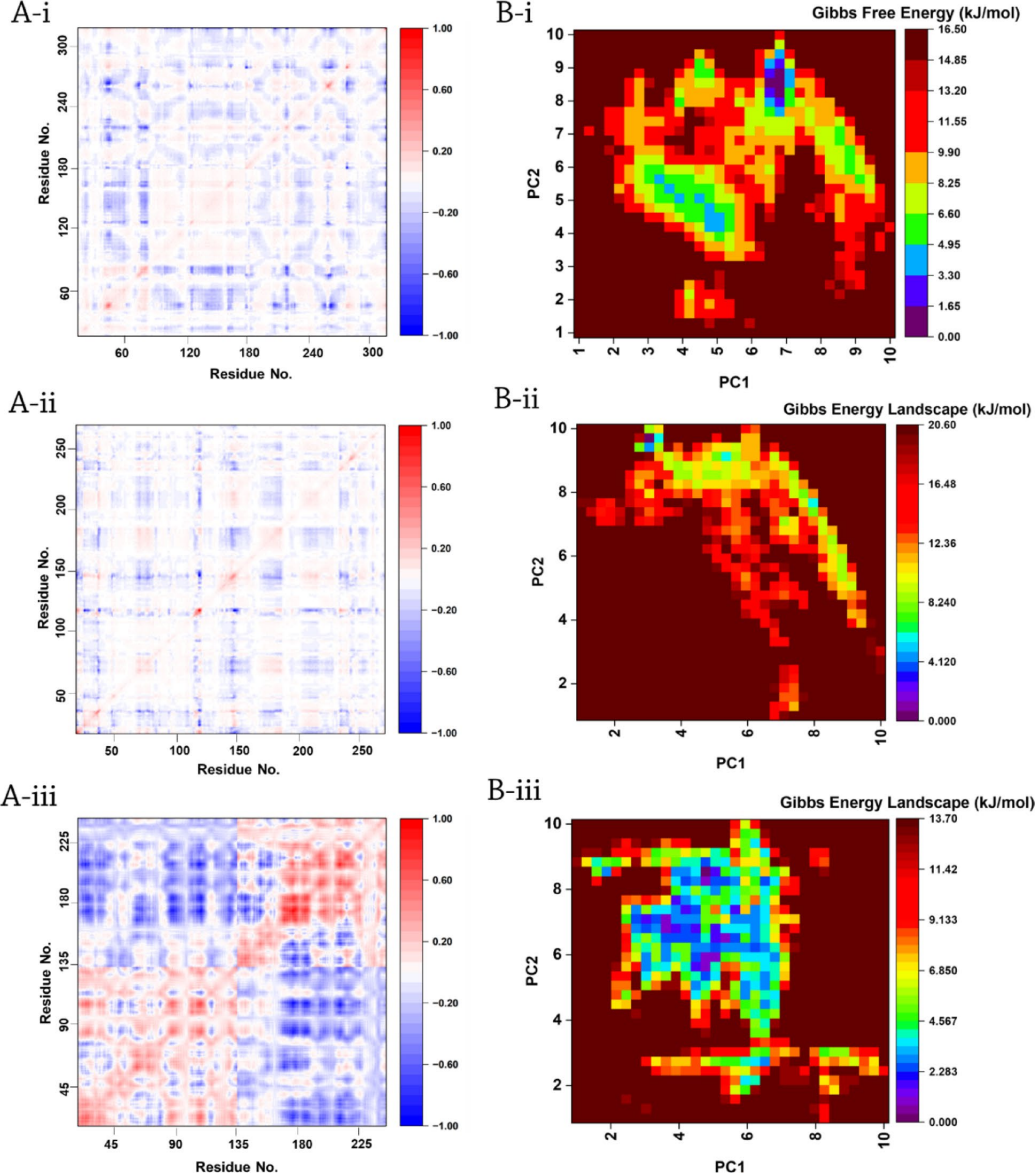
**A **Dynamic Cross-Correlation Matrix (DCCM) analysis of **i** *PTEN*/Enzalutamide, **ii** *MAG*/GT1b, and **iii** *NOTCH1*/Crenigacestat complexes. **B **Free energy landscape (FEL) analysis of **i** *PTEN*/enzalutamide, **ii** *MAG*/GT1b, and **iii** *NOTCH1*/Crenigacestat complexes.

The dynamical properties of the *PTEN*/enzalutamide complex were investigated using DCCM analysis to elucidate potential mechanisms relevant to axonal regeneration (Fig. 5A-i). The anti-correlation (covariance =  −1) suggests that specific residues exhibit opposing displacements during simulation, potentially reflecting enzalutamide-induced conformational adjustments or allosteric communication. As PTEN is a key regulator of the *PI3K/AKT* pathway, these dynamical changes could modulate its phosphatase activity or interactions with downstream signaling molecules critical for neuronal growth [50]. For instance, anti-correlated motions involving PTEN’s catalytic domain might alter its suppression of *AKT* signaling, a pathway essential for axon outgrowth.

Parallel DCCM analysis of the MAG/GT1b complex (Fig. 5A-ii) revealed distinct dynamical signatures, with strongly correlated motions (covariance up to 1) between residues implicated in GT1b recognition. In contrast, weaker correlations (e.g., −1) suggest minimal coordination in other regions, highlighting the specificity of MAG-GT1b interactions. Furthermore, in the NOTCH1/Crenigacestat system (Fig. 5A-iii), covariance patterns indicate ligand-stabilized dynamics that may perturb *NOTCH1* signaling—a pathway increasingly linked to axonal regeneration [51]. Together, these analyses demonstrate how small molecules (enzalutamide, Crenigacestat) and glycolipids (GT1b) induce unique dynamical perturbations in their respective targets, with potential cascading effects on axon repair mechanisms.

The FES analysis of PTEN in complex with enzalutamide reveals that enzalutamide binding stabilizes a low-energy basin (0–5 kJ/mol) corresponding to *PTEN*’s auto-inhibited state, while significantly raising the energy barrier (~16.5 kJ/mol) for transitions to membrane-associated conformations (Fig. 5B-i). This suggests that enzalutamide may impair PTEN’s ability to localize at growth cones or interact with axon guidance cues, a process essential for regenerative signaling. The drug’s allosteric suppression of PTEN’s open states could disrupt its phosphatase-dependent regulation of *PIP3*, a key mediator of neurite outgrowth [52]. These findings provide a thermodynamic and structural explanation for how enzalutamide might antagonize axon repair processes, which emphasizes the need to evaluate anti-androgen therapies in nerve injury contexts.

For the MAG/GT1b complex, the FEL derived from principal components PC1 and PC2 (Fig. 5B-ii) features a pronounced global energy minimum at 20.6 kJ/mol, reflecting the high-affinity binding between MAG and its glycolipid ligand GT1b. The depth of this basin suggests strong electrostatic and hydrophobic complementarity, while multiple local minima highlight conformational plasticity that may enable functional versatility in neuronal signaling. These results support targeting the MAG/GT1b interaction to modulate axon growth inhibition for therapeutic development.

In the case of NOTCH1/Crenigacestat, the FEL (Fig. 5B-iii) shows a deep energy minimum (13.7 kJ/mol), indicating thermodynamic stabilization of NOTCH1 upon inhibitor binding. The observed metastable states along PC1 and PC2 suggest that Crenigacestat induces conformational changes that may lock NOTCH1 into an inhibitory state, potentially modulating γ-secretase activity. This provides a mechanistic basis for investigating Crenigacestat as a candidate in axonal regeneration therapies.

#### Polymeric nanocarriers for delivering siRNAs and drugs

An important factor in determining the stability of nanocarriers is their interactions and impact on one another. In this regard, we have investigated binary interactions, including polymers/siRNA and polymers/drugs, as well as their impact when they are simulated together. The interactions are evaluated through the course of simulations, and the average energies are calculated and discussed in this section (Figure S2). At first glance, it can be observed that among all simulations, chitosan-based nanocarriers have the strongest interactions with siRNAs, where the electrostatic energy contributed mostly to the observed interactions.

**Fig. 6:**
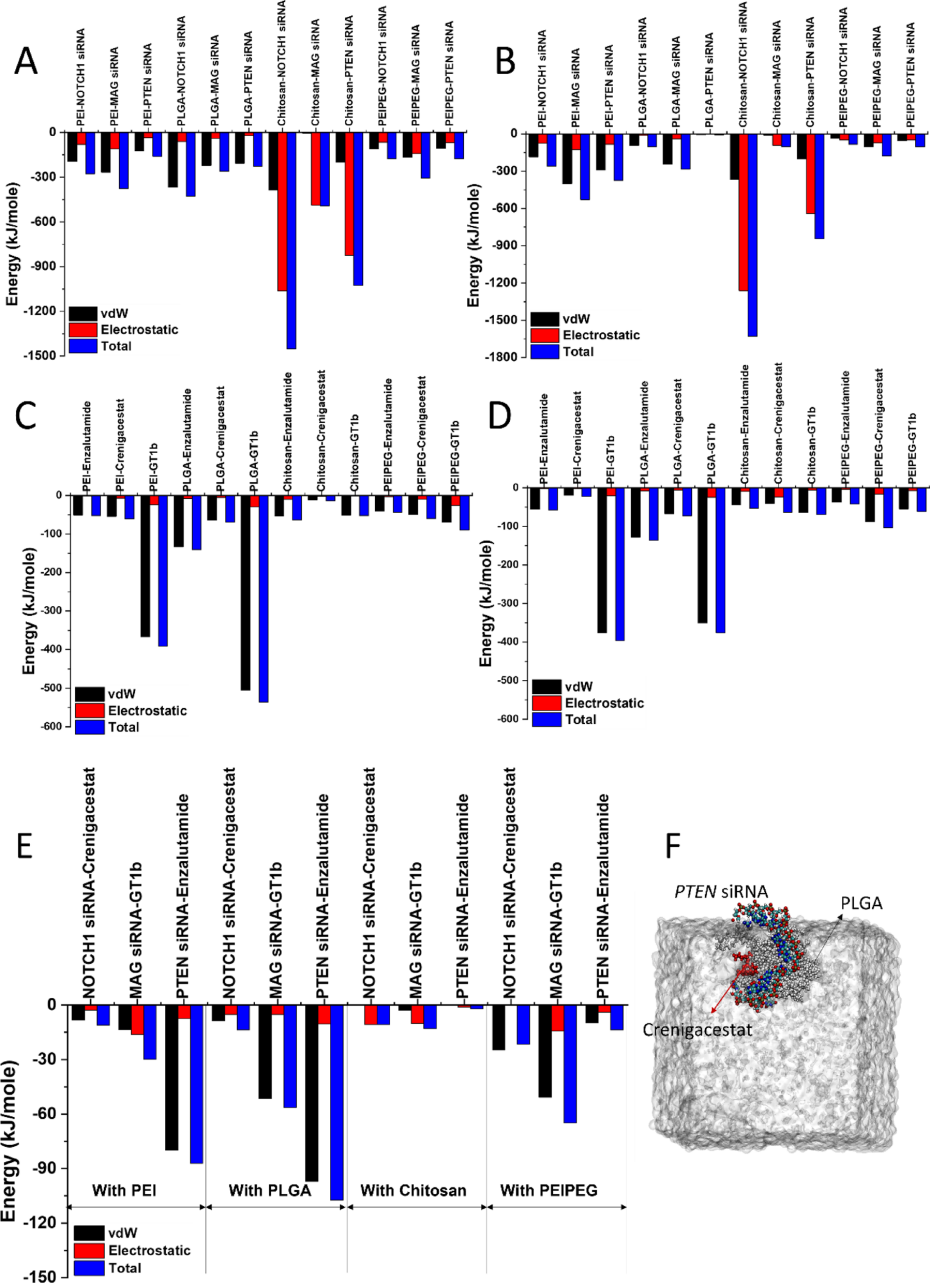
Interaction energy analysis. (**A**, **B**) the energy of interactions between polymers/siRNAs at binary simulations and in the presence of drugs, respectively. (**C, D)** the energy of interactions between polymers/drugs at binary simulations and in the presence of siRNAs, respectively. (**E)** The energy of drug/siRNA interactions in the third set of simulations, (**F**) Snapshot of the PLGA/*PTEN*/Crenigacestat simulation. (vdW=Van der Waals)

According to Fig. 6A, the binary interactions of siRNAs with polymers show that chitosan-NOTCH1 siRNA exhibits the strongest interaction ( −1451.74 kJ/mol) among its peers, which is mainly based on electrostatic interactions ( −1064.60 kJ/mol). In addition, chitosan-PTEN siRNA also exhibited strong interaction with  −1025.78 kJ/mol total attraction, with its greater portion being similarly electrostatic energy ( −825.13 kJ/mol). Figure 6B depicts the same interactions between polymers and siRNAs in the presence of drugs. Similarly, chitosan and siRNAs exhibit a similar trend, with the strongest interaction corresponding to chitosan-NOTCH1 siRNAs. It was interesting to observe that the presence of drugs in each composition enhanced the attraction between chitosan and siRNA as the total energy increased to  −1630.02 kJ/mol. However, the PLGA-PTEN pair exhibited weakened attraction when enzalutamide was added to the simulation box, as the attraction dropped from  −227.67 kJ/mol to  −6.88 kJ/mol when enzalutamide was introduced to the nanocarrier’s composition.

Later, the interaction of drugs with their polymeric carriers with and without siRNA was examined in Fig. 6C and D, respectively. It is worth noting that similar trends are observed between the energies between polymers and drugs in both approaches, which indicates their repeatability and, therefore, their reliability. Moreover, it should be noted that, although chitosan exhibited favorable interaction energies with siRNAs, PLGA and PEI showed more favorable interaction energies with drug molecules, particularly GT1b, as depicted in Fig. 6C, suggesting a higher drug affinity and encapsulation potential.

In simulations with all three components (Figure S3), we should consider the interactions between drugs and siRNAs, which are presented in Fig. 6E. The interactions between drugs and siRNAs are minimal compared to their interactions with polymers individually. This difference can be attributed to the fact that vdW energy constitutes most of the calculated total energy. The strongest interactions between siRNAs occur with PTEN siRNA-enzalutamide in the presence of PLGA (−107.31 kJ/mol) and PEI (-87.15 kJ/mol), primarily driven by vdW forces. SiRNAs show a greater affinity for chitosan polymer chains than for drug molecules. In contrast, PLGA and PEI exhibit a stronger tendency to interact with the drug molecules. As illustrated in Fig. 6F, the imulation reveals a minimal interaction between the PLGA/PTEN/Crenigacestat drug and the siRNA structure, indicating a negligible loading into the polymeric assembly. This observation validates the presence of only fragile interactions between PLGA/PTEN and PLGA/Crenigacestat.

#### Polymer chain properties in simulations

**Fig. 7:**
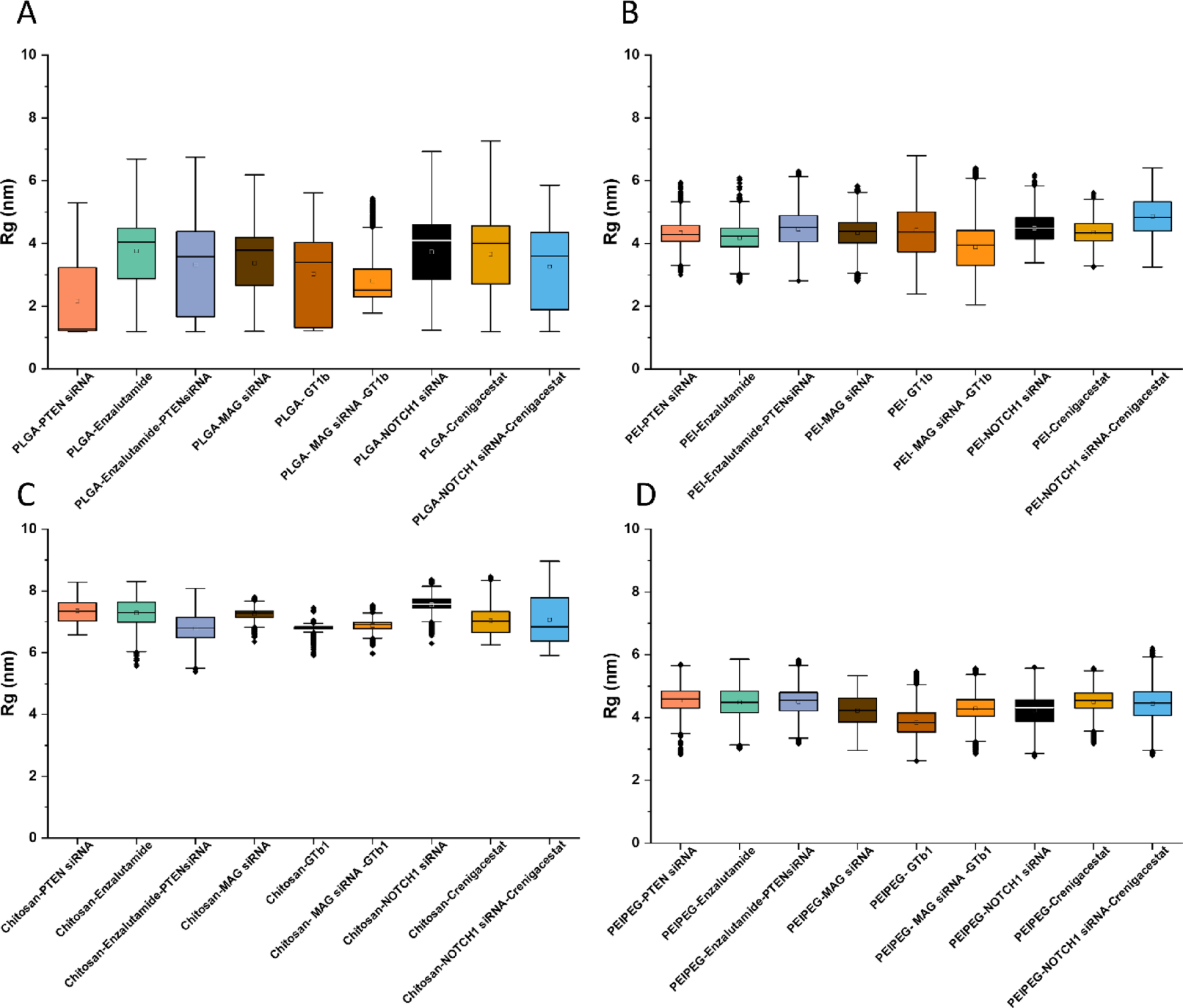
**A–D** The distribution of calculated Rg for PLGA-, PEI-, chitosan-, and PEI-PEG-based simulation systems, respectively.

The radius of gyration (Rg) of polymer chains is calculated during the simulation time (Figure S4). To gain insight into the results, Fig. 7A-D show the distributions of calculated Rg for PLGA, PEI, chitosan, and PEI-PEG chains, respectively. PLGA-based systems (Fig. 7A) exhibit a broader Rg distribution compared to other polymers, which can be attributed to their amphiphilic nature. Upon hydration, PLGA strands undergo rapid self-assembly but frequently form multiple small, dispersed nanocarriers rather than a single consolidated structure. This heterogeneous assembly behavior results in the observed polydispersity in Rg values. In contrast, PEI-based nanocarriers (Fig. 7B) display a narrower Rg distribution with a larger mean value. This homogeneity reflects the strong hydrophilicity of PEI, which promotes the formation of a single, well-defined nanocarrier rather than fragmented assemblies. The increased Rg correlates with the energy analysis presented earlier, where PEI demonstrated lower affinity for siRNA and drug molecules, resulting in more extended, loosely packed structures.

Chitosan-based systems (Fig. 7C) exhibit the largest Rg values among all polymers studied. This arises from the polymer's intrinsic structural properties: (1) the bulky aromatic rings in chitosan's backbone restrict chain flexibility, and (2) the extended conformation of the semi-rigid polymer strands leads to more voluminous assemblies. PEI-PEG nanocarriers (Fig. 7D) show intermediate behavior, mirroring the trends observed for PEI due to their structural similarity, albeit with slightly modulated assembly characteristics from PEG incorporation. These observations highlight the complicated relationship among multiple factors governing nanocarrier formation, including polymer hydrophobicity/hydrophilicity balance, molecular rigidity and chain flexibility, competitive interactions between polymer-drug-siRNA components, and solvation dynamics during self-assembly.

**Fig. 8:**
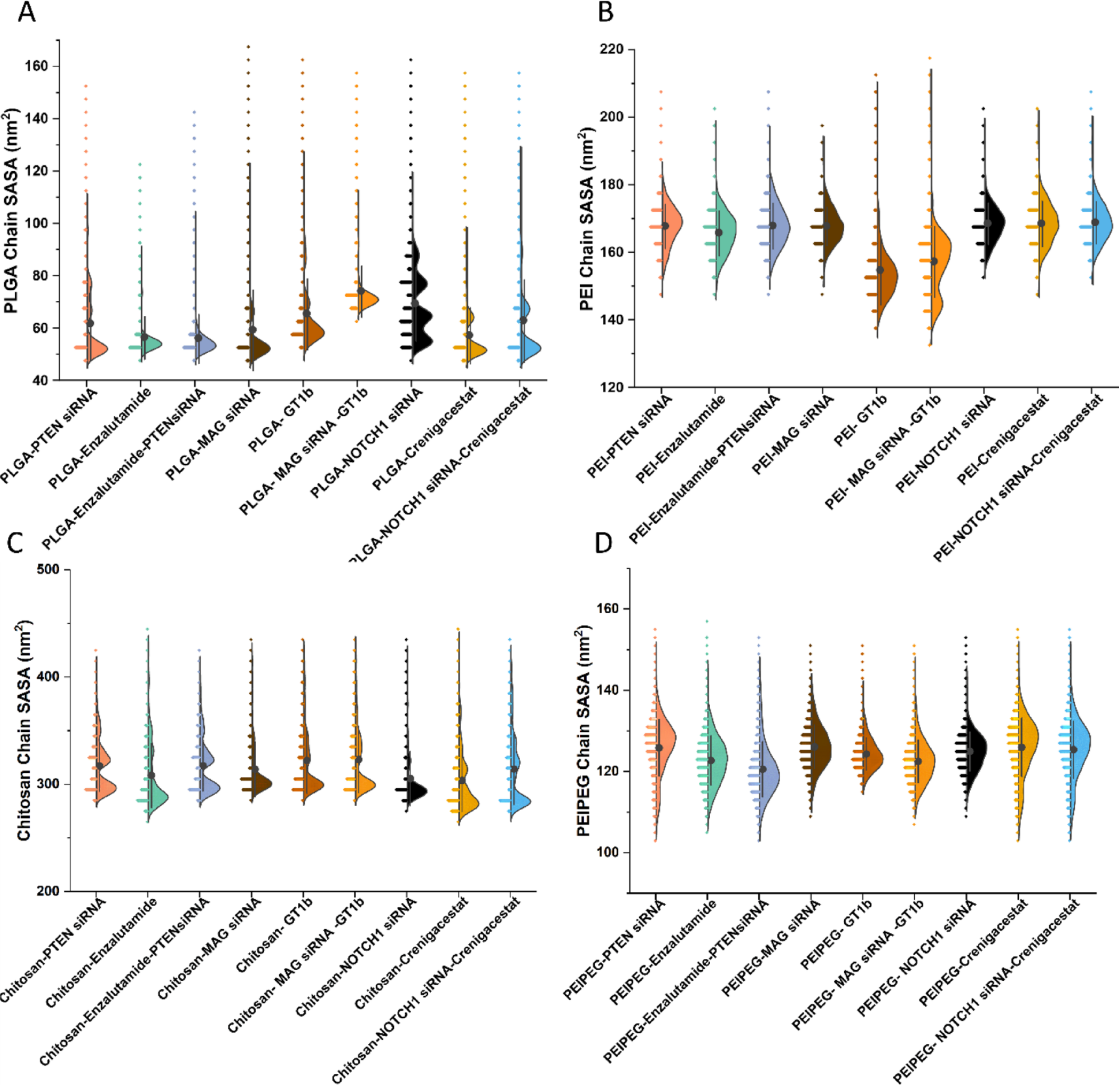
**A–D** PLGA, PEI, chitosan, and PEI-PEG chains’ SASA for various interactions of drugs/polymers/siRNAs, respectively. (SASA=Solvent-Accessible Surface Area)

The SASA analysis of molecules is influenced by elements such as the polymer ratio and the hydrophobicity or hydrophilicity of the molecules. Reduced SASA values are indicative of a hydrophobic polymer structure, which tends to aggregate and form assemblies rather than extending toward water molecules (Figure S5). Figure 8A-D depict the distribution and mean SASA values of the polymers in individual simulations. It has been observed that PLGA polymer chains exhibit reduced average SASA values (approximately 40–90 nm^2^) in comparison with PEI chains (approximately 130–180 nm^2^) and PEI-PEG (approximately 100–150 nm^2^). On the other hand, chitosan-based systems exhibited the highest range of SASA (approximately 270–320 nm^2^) which is due to their bigger strands and hydrophilic nature.

## Discussions

Several studies have investigated the application of network analysis methods to identify genetic loci associated with axonal regeneration [53, 54]. In addition, network analysis methods have been used to identify genes with significant correlation with SCI-associated pathways and sequelae in multiple studies [55–58]. The results of such studies have implicated various genes in post-SCI degenerative processes [59–62]. The purpose of this study is to investigate the potential of small molecules and siRNA-mediated modulation of cellular processes in SCI regeneration.

In this study, a systems biology approach was applied to identify genetic loci negatively associated with axonal regeneration and oligodendrocyte differentiation in SCI. GO analysis and systems biology approaches were conducted on the data retrieved from various relevant databases and literature (Tables S1 and S2). Further PPI analysis and identification of hub genes, as well as identification of the genes negatively regulating spinal cord regeneration, ultimately led to the finding of the three genetic loci MAG, negatively associated with axonal regeneration, and PTEN and NOTCH1, which negatively affect the regenerative processes in SCI by affecting oligodendrocyte differentiation.

This study illustrates a novel computational method that integrates gene network analysis, siRNA design, and small-molecule docking with molecular dynamics simulations to evaluate delivery strategies targeting key regulators of spinal cord injury. Specifically, we present the first comparative analysis of PLGA, PEI, chitosan, and PEI-PEG nanocarriers for the co-delivery of siRNAs and small molecule inhibitors against *MAG*, *PTEN*, and *NOTCH1*. These genes have previously been studied individually, but this study is a first in conducting such methods in a unified regenerative strategy on this set of genetic loci by means of dual modality targeting.

According to existing literature, there is a significant potential for synergy between siRNA and small-molecule delivery by means of various mechanisms, including small-molecule-mediated endosomal escape of siRNAs, bioconjugation of siRNA to small molecules, which can in turn reduce their toxicity and immunogenicity and enhance their efficacy, and incorporation of small molecules into nanocarriers, which improves siRNA stability and delivery [63–67]. Unlike earlier studies that examined isolated elements of delivery or gene inhibition, our approach simultaneously evaluates the target engagement, molecular stability, and carrier compatibility *in silico*. These findings offer preliminary insights that could guide future experimental investigations.

However, it should be kept in mind that while this study focused on three key negative regulators, based on their roles in spinal cord regeneration, we acknowledge that this limited gene set does not fully showcase the complexity of the regenerative processes. Potential interactions between other regulatory genes, or the effects of simultaneous effects on multiple pathways, are currently unexplored. Therefore, future studies incorporating combination targeting strategies and a wider range of gene candidates can provide a deeper understanding of SCI repair mechanisms and their therapeutic implications.

MAG, expressed by myelination-associated glial cells, is a myelin inhibitor that is a regulator of axon growth with an age-dependent effect, inducing this process during developmental stages and inhibiting it in adult neurons [68]. Although several *in vivo* studies have indicated that the deletion of this genetic locus does not induce axonal regeneration, the lack of sufficient data and limited number of studies in this regard call for the conduction of further research to unravel the true role of MAG in CNS regeneration [12].

The second locus identified in this study is PTEN, a negative regulator of the *PI3K* signaling pathway, which is associated with CNS myelination and differentiation of the oligodendrocyte progenitor cells [69]. In a study by Walker *et al*. [70], bisperoxovanadium-mediated inhibition of PTEN significantly contributed to the protection of oligodendrocytes and preservation of the white matter in a model of rat SCI. Lastly, the final identified locus, NOTCH1, encodes a component of the *NOTCH* signaling pathway, which has been associated with glial differentiation and inhibition of neuronal differentiation [71, 72]. According to the results of several studies, the inhibition of NOTCH1 leads to the induction of neuronal differentiation, and accordingly, functional improvement in models of SCI [73–75].

Although multiple genes are known to play a role in oligodendrocyte differentiation and axonal regeneration, MAG, PTEN, and NOTCH1 were particularly chosen for this investigation based on three important factors: They are important hub genes in the networks of axonal regeneration and oligodendrocyte differentiation because of their high degree centrality as determined by Cytoscape network analysis; their functional validation by GeneMANIA and Enrichr, which verified their involvement in negative regulation of processes related to neural regeneration; and their established relevance in the scientific literature as regulators of myelination, neurogenesis, and glial differentiation. They were prioritized as representative targets for siRNA evaluation and drug delivery through polymeric nanocarriers because of these factors taken together [76].

Furthermore, in this study, the *in silico* targeting of these genes by means of RNA interference and pharmaceutical agents was conducted. Three potential siRNAs were designed with the highest specificity and least off-target alignment to conduct RNA interference (Table 1, Figure 1A). It should be considered that although the siRNA candidates were designed using established prediction algorithms and filtered for sequence specificity and structural stability, their efficacy remains to be validated experimentally. The *in vivo* efficacy of such agents is largely affected by factors such as method of delivery, endosomal escape and disassembly of siRNAs after their cellular uptake, and secondary structures of mRNA molecules, which can negatively affect their interaction [77, 78].

Moreover, although this study aimed to minimize off-target binding of siRNAs, it remains a source of possible adverse events. siRNAs can cause off-target gene silencing effects due to partial complementarity to unintended mRNA sequences, which, in turn, can lead to complications in interpreting the effects of their application and potential unwanted toxicity [79, 80]. In addition, three pharmaceutical agents, Crenigacestat [81], GT1b, and Enzalutamide [82], targeting NOTCH1, MAG, and PTEN, respectively, were used in the MD simulation (Table 3). Crenigacestat and enzalutamide were determined through the drug-gene database. On the other hand, GT1b is a brain ganglioside, which is a site of attachment for MAG in the nervous system involved in axon-myelin interactions [83]. Such interactions between this agent and MAG were the basis of its implication as a competitive inhibitor of *MAG*.

The results of the MD simulations indicated that MAG and PTEN had more favorable interactions with their corresponding drugs and siRNAs in comparison with NOTCH1. The differential interaction profiles observed in this study may be due to the structural and functional characteristics of the NOTCH1 and MAG proteins. NOTCH1 is a large, multidomain transmembrane receptor with a heavily glycosylated extracellular domain involved in highly regulated signal transduction pathways, often requiring specific ligand-induced conformational changes and proteolytic cleavage for activation [84, 85]. As a result of such features, small molecules, such as Crenigacestat, may have limited ability to access or bind effectively to NOTCH1 under idealized MD simulation conditions, especially since such simulations may not take into account the induced-fit mechanisms or the dynamic conformational changes required for activation [84, 86].

In contrast, MAG is a peripheral myelin protein with well-defined carbohydrate-recognition domains that facilitate specific interactions with gangliosides like GT1b. Moreover, MAG’s extracellular domain provides a more accessible and stable binding interface in the simulated aqueous environment, favoring a tighter and sustained association with GT1b [87, 88]. These intrinsic features of the protein are likely responsible for the observed interaction behavior results. It should be considered that targeting broadly acting genes such as PTEN and NOTCH1*,* which are involved in many cellular signaling pathways and developmental processes, can lead to off-target effects and, as a result, adverse outcomes. For instance, targeting these genes can, in turn, alter the expression of the associated genes and cause potential side effects that might not be easily interpreted. In addition, manipulation of such loci can negatively affect critical physiological processes, raising concerns regarding the safety of these interventions upon clinical application [89–91].

The delivery of the siRNAs and drugs targeting the gene candidates by polymeric bioresponsive nanocarriers was assessed using MD simulations. MD simulations can be applied in regenerative medicine studies in many areas. Such measurements can be used to predict the nature of interactions of cells with specific scaffolds or external stimuli and can help us analyze molecular-level formation and optimization of substrates. For instance, we can assess the binding strength of biomolecules to materials and the effects of such interactions on biological activity [92]. In addition, such simulations can assist with optimization of delivery systems for gene therapy purposes by means of assessing the dynamic assembly and behaviors of such systems when interacting with biological components [93].

However, MD simulations rely on several idealized conditions, including utilization of classical force fields, which in turn will not capture the quantum effects and lead to reduced accuracy. In addition, the time and size scales are limited, which will cause insufficient sampling [94, 95]. Since MD simulations are limited to modeling at an atomic level over short times, they do not represent the complexities of the biological environment, including the formation of protein coronas around nanoparticles and the processes involved in the blood-brain barrier (BBB) penetration [96, 97]. Furthermore, these simulations utilize fixed and uniform ionic strength, which does not represent the dynamic and spatially heterogeneous *in vivo* conditions [98, 99]. Protein folding modeling in MD simulations often involves modeling this process in dilute solutions without chaperones or other important key factors that are normally involved in *in vivo* conditions [100, 101]. Lastly, such simulations typically don’t consider degradation processes, which play an important role in the overall picture of protein fate *in vivo* [100, 102]. Therefore, although MD simulations are powerful computational tools for studying the behavior of molecules at the atomic level, these idealized conditions cause important limitations in their overall accuracy and predictive power.

Regarding the polymers used, PEI is extensively used in preclinical studies. In a study by Ding *et al*. [103], PEI-alginate nanoparticles were used as a delivery system for Nischarin-targeting siRNA in a rat model of SCI. The results of this study provide preliminary evidence of the positive effects of this method of delivery, both in levels of gene expression by significantly inhibiting Nischarin expression and in the functional assessment of the treatment group in comparison with controls [103]. PEI was also used in the delivery of plasmid DNA to the rat spinal cord, which showed 40 times higher transgene expression in comparison with the naked plasmid [104].

In a study by Imanpour *et al*. [105], RNA interference of specific genes negatively regulating bone healing was simulated by comparing three polymers: PEG, PEI, and PEG/PEI copolymer. The results of this study showed that delivery of the hsa-mIR-146a-5p with PEG-PEI was the most stable. In addition, PEI can be a candidate for delivering hsa-mIR-7155 [105]. Another study on diabetic wound healing used three polymers, including PLGA, PEI, and chitosan, in the MD simulation, among which the PLGA/hsa-mir-422a showed the most stable integration [106]. Results of such studies demonstrate preliminary evidence that PEI has advantages as a drug delivery system.

However, there are some limitations in applications of these nanocarriers at the bedside. There is a risk of cytotoxicity, especially at higher concentrations or molecular weights of unmodified PEI [107, 108]. Furthermore, poor biodistribution and low targeting efficacy in studies with *in vivo* methods result in off-target accumulation, especially in cases of systemic administration [109]. There is also an issue with stability in biological environments, which can result in degradation or loss of function [110].

To overcome such limitations, several optimization strategies exist for PEI-based carriers to utilize them in future experimental studies. Application of low-molecular-weight or linear PEI has been shown to reduce the overall cytotoxicity [111]. Also, low-molecular-weight PEI cross-linked with disulfide bonds enables intracellular release and is a more biocompatible option [112, 113]. Regarding the limitations in efficacy, lipid or fluorine substitution can improve targeted delivery, serum stability, and cellular uptake [111, 114]. PEG can improve DNA material aggregation in the scaffold, which can be limited by reduced uniformity and delivery efficacy [110]. Biomimetic approaches such as coating the carriers with natural cell membranes can also reduce immunogenicity [115]. In addition, strategies such as local delivery, tailoring biochemical characteristics of the carrier according to the target tissue, and pharmacokinetic improvement can enhance the overall administration and biodistribution efficacy [114, 116].

More specifically, scaffolds made from PEI used in the treatment of CNS conditions show poor BBB penetration, which in turn affects the efficacy of delivering substances to the target tissue [117, 118]. Several approaches, including attachment of specific ligands, hydrophobic modifications, intranasal delivery, combination with other polymers, and use of external stimuli such as magnetic fields, can be used to address this problem [119, 120].

In this study, we aimed to evaluate polymeric carriers for the co-delivery of siRNA and small-molecule drugs targeting key inhibitors of spinal cord regeneration. Our *in silico* findings suggest that among the delivery systems modeled, PLGA demonstrated superior stability and compatibility for transporting both therapeutic modalities. These results suggest PLGA as a possible candidate for combinatorial gene and drug delivery strategies in SCI. Overall, this study provides an insight into the genetic and molecular characteristics of the SCI repair processes and the potential therapeutic agents targeting such components. Nevertheless, further preclinical and clinical studies are necessary in this matter to assess their efficacy and safety in cases of SCI.

Because of its controlled and sustained drug release properties, PLGA stands out among the polymers under investigation. Depending on its lactic-to-glycolic acid ratio, it can release therapeutic agents over a period of days to weeks. PEI, on the other hand, has a high cationic charge, which makes it more efficient for delivering genes. However, when used systemically, it usually necessitates additional surface modifications to cross the BBB. Future *in vivo* studies that seek to confirm the sustained release potential of PLGA and assess the therapeutic efficacy of localized nanocarrier delivery in SCI models will address these issues, even though we acknowledge that our *in silico* simulations do not model BBB transport and drug release kinetics over time.

## Conclusions

This work included the *in silico* targeting of the products of MAG, PTEN, and NOTCH1 genes using both RNA interference and pharmacological drugs. Three possible siRNAs were engineered to exhibit maximal specificity and little off-target alignment for the purpose of RNA interference. Furthermore, three pharmacological agents—Crenigacestat, GT1b, and Enzalutamide—targeting NOTCH1, MAG, and PTEN, respectively, were used in the MD simulation. Crenigacestat and enzalutamide were identified using the drug-gene database. Conversely, GT1b is a brain ganglioside that serves as a binding site for *MAG* in the nervous system, facilitating axon-myelin interactions. The interactions between this drug and *MAG* underpinned its designation as a competitive MAG inhibitor.

The efficacy of polymeric bioresponsive nanocarriers in delivering siRNAs and drugs aimed at products of the gene candidates was evaluated using molecular dynamics simulations. The findings of this investigation serve as a foundation for identifying potential therapeutic targets to promote regeneration, particularly in central nervous system injuries and specifically in spinal cord injuries. Notably, the study provides insights into the potential use of specific inhibitors for genes involved in negative regulation of regeneration.

Moreover, this study focused on computational optimization of nanocarriers and therapeutic agent encapsulation using MD simulations. While our approach provides valuable insights into polymer-drug/siRNA interactions and encapsulation efficiency, it specifically examines the pre-biological formulation stage under idealized conditions. The simulations do not account for physiological complexities such as protein corona formation, interactions with components of the respective physiological processes, or *in vivo* targeting efficiency, as these phenomena require fundamentally different computational approaches and experimental validation. These aspects represent important next steps in nanocarrier development beyond the current scope, which was designed to establish foundational parameters for effective nanocarrier formulation prior to biological testing. The findings should therefore be interpreted as a crucial first step in nanocarrier design, with subsequent biological evaluation being necessary for translational applications. The next steps involve the identification of the best siRNAs and small molecules to further explore therapeutic options.

## Electronic supplementary material

Supplementary material

## Data Availability

All data and materials are available from the corresponding author.

## Abbreviations

AA-MD: All-atom MD
BBB: Blood-brain barrier
CNS: Central nervous system
DAVID: Database for annotation, visualization, and integrated discovery
DCCM: Dynamic cross-correlation matrix
ECM: Extracellular matrix
FEL: Free energy landscape
GO: Gene ontology
GBD: Global burden of disease
H-bonds: Hydrogen bonds
MD: Molecular dynamics
MM-PBSA: Molecular Mechanics Poisson-Boltzmann Surface Area
MAG: Myelin-associated glycoprotein
NOTCH1: Neurogenic locus notch homolog protein 1
OPCs: Oligodendrocyte progenitor cells
PME: Particle Mesh Ewald
PTEN: Phosphatase and tensin homolog
PI3K: Phosphatidyl inositol 3-kinase
PLGA: Poly (lactic-co-glycolic acid)
PEG: Polyethylene glycol
PEI: Polyethylene imine
PCA: Principal component analysis
PDB: Protein data bank
PPI: Protein-protein interaction
STRING: Search tool for the retrieval of interacting genes
SASA: Solvent-accessible surface area
SASA: Solvent-available-surface-area
SCI: Spinal cord injury
SCI: Spinal cord injury
SD: standard deviation
VMD: Visual molecular dynamics
YLD: Years lived with disability
