# Supplementary material for: Unraveling the role of MAG, PTEN, and NOTCH1 in axonal regeneration: a network analysis and molecular dynamics study of siRNA/drugs/nanocarriers interactions
Source: J Transl Med. 2025 Oct 1;23:1038. doi: 10.1186/s12967-025-07042-9 (PMC12487255; doi:10.1186/s12967-025-07042-9)
Supplement: Supplementary file 1 — Supplementary material [file 12967_2025_7042_MOESM1_ESM.docx]

Unraveling the Role of MAG, PTEN, and NOTCH1 in Axonal Regeneration: A Network analysis and Molecular Dynamics Study of siRNA/drugs/nanocarriers Interactions

Alireza Salimi^1^, Aysan Moeinafshar^2, 3^, Sima Rezvantalab^4**^, Mohammad Dabiri^5^, Nima Rezaei^6, 7, 8^, Nima Beheshtizadeh^9, 10, 11*^

*1. Roy J. and Lucille A. Carver College of Medicine, University of Iowa, Iowa City, IA 52242, USA.*

*2. School of medicine, Tehran University of Medical Sciences, Tehran, Iran*

*3. Regenerative Medicine Group (REMED), Universal Scientific Education and Research Network (USERN), Tehran, Iran.*

*4. Chemical Engineering Department, Urmia University of Technology, Urmia 57166‑419, Iran*

*5. Department of Molecular Biology, Faculty of Natural Sciences, Comenius University in Bratislava, Ilkovičova 6, 841 04 Bratislava, Slovak Republic*

*6. Department of Immunology, School of Medicine, Tehran University of Medical Sciences, Tehran, Iran*

*7. Research Center for Immunodeficiencies, Children’s Medical Center, Tehran University of Medical Sciences, Tehran, Iran*

*8. Network of Immunity in Infection, Malignancy and Autoimmunity (NIIMA), Universal Scientific Education and Research Network (USERN), Tehran, Iran*

*9. Department of Tissue Engineering, Faculty of Advanced Medical Sciences, Tabriz University of Medical Sciences, Tabriz, Iran*

*10. Stem Cell Research Center, Tabriz University of Medical Sciences, Tabriz, Iran*

*11. Tabriz USERN Office, Universal Scientific Education and Research Network (USERN), Tabriz, Iran*

Corresponding authors:

* Nima Beheshtizadeh (email: n.beheshtizadeh@tbzmed.ac.ir)

** Sima Rezvantalab (s.rezvantalab@uut.ac.ir)

**Table S1**. The list of 285 genes participated in the CNS regeneration processes gathered from regeneration gene database

| **No.** | **Gene Name** | **Entrez ID** |
| --- | --- | --- |
|  | STX12 | 23673 |
|  | ADCYAP1, PACAP | 116 |
|  | AGER | 177 |
|  | AKT1 | 207 |
|  | AKR1B1 | 231 |
|  | APOA4 | 337 |
|  | APOD | 347 |
|  | GJC3 | 349149 |
|  | ARTN | 9048 |
|  | ATF3 | 467 |
|  | NEUROD6 | 63974 |
|  | BAX | 581 |
|  | BCL2 | 596 |
|  | BDNF | 627 |
|  | TSPO | 706 |
|  | CTNNB1 | 1499 |
|  | CDKN1A | 1026 |
|  | CEBPB | 1051 |
|  | CEBPD | 1052 |
|  | CHL1 | 10752 |
|  | SOCS3 | 9021 |
|  | CLU | 1191 |
|  | CRABP2 | 1382 |
|  | CREBBP | 1387 |
|  | VCAN | 1462 |
|  | DHFR | 1719 |
|  | EGFR | 1956 |
|  | EPHA4 | 2043 |
|  | FGF2 | 2247 |
|  | FGFR1 | 2260 |
|  | FLOT2 | 2319 |
|  | TLR3 | 7098 |
|  | GAP43 | 2596 |
|  | GFAP | 2670 |
|  | GFRA3 | 2676 |
|  | GJB1 | 2705 |
|  | LRP2 | 4036 |
|  | HDAC5 | 10014 |
|  | HN1 | 51155 |
|  | NDST1 | 3340 |
|  | ICAM1 | 3383 |
|  | ID2 | 3398 |
|  | IFRD1 | 3475 |
|  | IL17A | 3605 |
|  | IL6 | 3569 |
|  | ITGA7 | 3679 |
|  | ITGB1 | 3688 |
|  | J U N | 3725 |
|  | KIF3C | 3797 |
|  | KLF4 | 9314 |
|  | KLF9 | 687 |
|  | L1CAM | 3897 |
|  | LGALS1 | 3956 |
|  | LGALS3 | 3958 |
|  | LIF | 3976 |
|  | LPL | 4023 |
|  | SMAD1 | 4086 |
|  | MAG | 4099 |
|  | MATN2 | 4147 |
|  | STX12 | 23673 |
|  | MBP | 4155 |
|  | MDK | 4192 |
|  | MMP2 | 4313 |
|  | MMP9 | 4318 |
|  | MPZ | 4359 |
|  | MT3 | 4504 |
|  | MYT1 | 4661 |
|  | NDRG1 | 10397 |
|  | NEUROD1 | 4760 |
|  | NEFL | 4747 |
|  | NGFR | 4804 |
|  | NINJ1 | 4814 |
|  | NOTCH1 | 4851 |
|  | NRP2 | 8827 |
|  | NTN1 | 9423 |
|  | NTRK2 | 4915 |
|  | O MG | 4974 |
|  | P2RY2 | 5029 |
|  | KAT2B | 8850 |
|  | PIK3R1 | 5295 |
|  | LILRB3 | 11025 |
|  | PLAT | 5327 |
|  | PLAUR | 5329 |
|  | PMP22 | 5376 |
|  | PPARGC1A | 10891 |
|  | PTEN | 5728 |
|  | PTPRS | 5802 |
|  | PTPRZ1 | 5803 |
|  | RAC1 | 5879 |
|  | RELN | 5649 |
|  | RET | 5979 |
|  | CCL13 | 6357 |
|  | SEMA3A | 10371 |
|  | SEMA4D | 10507 |
|  | SEMA7A | 8482 |
|  | ST8SIA4 | 7903 |
|  | SLC12A2 | 6558 |
|  | SOX11 | 6664 |
|  | SOX17 | 64321 |
|  | SPP1 | 6696 |
|  | SPRR1A | 6698 |
|  | SDC1 | 6382 |
|  | EFEMP1 | 2202 |
|  | TLR4 | 7099 |
|  | TNC | 3371 |
|  | TP53 | 7157 |
|  | TP63 | 8626 |
|  | TTR | 7276 |
|  | ULK1 | 8408 |
|  | VAV2 | 7410 |
|  | VEGFA | 7422 |
|  | NRSN1 | 140767 |
|  | TICAM2 | 353376 |
|  | TLR2 | 7097 |
|  | RGMA | 56963 |
|  | FCGR3A | 2214 |
|  | FCGR3B | 2215 |
|  | MAP3K12 | 7786 |
|  | TNFRSF12A | 51330 |
|  | TNFRSF19 | 55504 |
|  | GIPR | 2696 |
|  | MIR138-1 | 406929 |
|  | MIR206 | 406989 |
|  | GSK3B | 2932 |
|  | MTOR | 2475 |
|  | VAV3 | 10451 |
|  | CEND1 | 51286 |
|  | ZBP1 | 81030 |
|  | STK25 | 10494 |
|  | RTN4R | 65078 |
|  | NRP1 | 8829 |
|  | APOA5 | 116519 |
|  | RTN4 | 57142 |
|  | MI124-1 | 406907 |
|  | NTF4 | 4909 |
|  | ERN1 | 2081 |
|  | NDEL1 | 81565 |
|  | SIRT1 | 23411 |
|  | NRG1 | 3084 |
|  | SKIL | 6498 |
|  | NFIL3 | 4783 |
|  | NEU3 | 10825 |
|  | CNTN1 | 1272 |
|  | RHOA | 387 |
|  | FABP5 | 2171 |
|  | EP300 | 2033 |
|  | SPDYA | 245711 |
|  | C6 | 729 |
|  | CALCB | 797 |
|  | EPOR | 2057 |
|  | ERBB2 | 2064 |
|  | B4GALT1 | 2683 |
|  | IL1B | 3553 |
|  | MOG | 4340 |
|  | MT1A | 4489 |
|  | NCAM1 | 4684 |
|  | REG3A | 5068 |
|  | REG3G | 130120 |
|  | PLCD1 | 5333 |
|  | PRPH | 5630 |
|  | RARB | 5915 |
|  | VAMP2 | 6844 |
|  | SYP | 6855 |
|  | TF | 7018 |
|  | THY1 | 7070 |
|  | MYO5A | 4644 |
|  | ADM | 133 |
|  | GIP | 2695 |
|  | APOA1 | 335 |
|  | STAT3 | 6774 |
|  | PDGFRA | 5156 |
|  | MAPK10 | 5602 |
|  | SMO | 6608 |
|  | BCAN | 63827 |
|  | DPYSL2 | 1808 |
|  | CRYBB2 | 1415 |
|  | GDNF | 2668 |
|  | PSAP | 5660 |
|  | ROCK2 | 9475 |
|  | FN1 | 2335 |
|  | PPARG | 5468 |
|  | CTNF | 1270 |
|  | IFNG | 3458 |
|  | IGF1R | 3480 |
|  | APOE | 348 |
|  | EPHB3 | 2049 |
|  | CARTPT | 9607 |
|  | GAL | 51083 |
|  | ARG1 | 383 |
|  | SMAD2 | 4087 |
|  | FOXO3 | 2309 |
|  | MICAL1 | 64780 |
|  | MAP1B | 4131 |
|  | SKP2 | 6502 |
|  | ERBB3 | 2065 |
|  | SHH | 6469 |
|  | VAMP3 | 9341 |
|  | KCNK3 | 3777 |
|  | FERMT1 | 55612 |
|  | LRP1 | 4035 |
|  | WNT3A | 89780 |
|  | GORAB | 92344 |
|  | NGF | 4803 |
|  | ITGA4 | 3676 |
|  | MDM2 | 4193 |
|  | SLIT2 | 9353 |
|  | EFNB3 | 1949 |
|  | PXN | 5829 |
|  | POSTN | 10631 |
|  | B4GALT5 | 9334 |
|  | MICAL3 | 57553 |
|  | DIXDC1 | 85458 |
|  | ZDHHC23 | 254887 |
|  | PALLD | 23022 |
|  | MICAL2 | 9645 |
|  | FLRT3 | 23767 |
|  | ELAVL4 | 1996 |
|  | CADM1 | 23705 |
|  | IL17RD | 54756 |
|  | MAPK3 | 5595 |
|  | PDLIM1 | 9124 |
|  | GPC1 | 2817 |
|  | NCAN | 1463 |
|  | TGFB1 | 7040 |
|  | ERBB4 | 2066 |
|  | RAPGEF3 | 10411 |
|  | RNH1 | 6050 |
|  | SPTAN1 | 6709 |
|  | CALU | 813 |
|  | RASD1 | 51655 |
|  | SLIT1 | 6585 |
|  | FAM168B | 130074 |
|  | MAPK14 | 1432 |
|  | CSPG4 | 1464 |
|  | ROCK1 | 6093 |
|  | UBE2B | 7320 |
|  | AKAP12 | 9590 |
|  | SLIT3 | 6586 |
|  | CDKN1B | 1027 |
|  | CIT | 11113 |
|  | GIPC1 | 10755 |
|  | ROBO2 | 6092 |
|  | NRTN | 4902 |
|  | CHST3 | 9469 |
|  | TRPC4 | 7223 |
|  | HDAC6 | 10013 |
|  | GRIA3 | 2892 |
|  | TRIM71 | 131405 |
|  | IFT43 | 112752 |
|  | PXDN | 7837 |
|  | MIRLET7A1 | 406881 |
|  | MYCBP2 | 23077 |
|  | AKT2 | 208 |
|  | SPAST | 6683 |
|  | PROK2 | 60675 |
|  | CDH2 | 1000 |
|  | CDH4 | 1002 |
|  | PAX6 | 5080 |
|  | TGIF1 | 7050 |
|  | TUBA1C | 84790 |
|  | HNRNPK | 3190 |
|  | MCAM | 4162 |
|  | CDKN2A | 1029 |
|  | RTN3 | 10313 |
|  | DOCK3 | 1795 |
|  | GATA6 | 2627 |
|  | IGF1 | 3479 |
|  | ITGA6 | 3655 |
|  | ITGB4 | 3691 |
|  | NEFH | 4744 |
|  | NTF3 | 4908 |
|  | GHRL | 51738 |
|  | NDE1 | 54820 |
|  | MAPK1 | 5594 |
|  | ACKR3 | 57007 |
|  | PLEKHB1 | 58473 |
|  | RIT1 | 6016 |
|  | SCN9A | 6335 |
|  | SET | 6418 |
|  | OCM | 654231 |
|  | BRAF | 673 |
|  | CASP2 | 835 |
|  | CASP6 | 839 |
|  | CAV1 | 857 |
|  | RHO | 6010 |

**Table S2**. The list of 76 genes participated in the CNS regeneration processes gathered from Literature

|  | | |
| --- | --- | --- |
| **No.** | **Gene Name** | **Entrez ID** |
| 1. | TGFA | 7039 |
| 2. | PLG | 5340 |
| 3. | HDAC1 | 3065 |
| 4. | HDAC2 | 3066 |
| 5. | HDAC3 | 8841 |
| 6. | HDAC4 | 9759 |
| 7. | HDAC8 | 55869 |
| 8. | MYC | 4609 |
| 9. | ASCL1 | 429 |
| 10. | IGF2 | 3481 |
| 11. | MIR9-1 | 407046 |
| 12. | LAMB2 | 3913 |
| 13. | HIF1A | 3091 |
| 14. | NREP | 9315 |
| 15. | HGF | 3082 |
| 16. | PAX2 | 5076 |
| 17. | PAX7 | 5081 |
| 18. | VIM | 7431 |
| 19. | SPARC | 6678 |
| 20. | DCN | 1634 |
| 21. | SRC | 6714 |
| 22. | PAX3 | 5077 |
| 23. | ITGAV | 3685 |
| 24. | RPS6KB1 | 6198 |
| 25. | CASP3 | 836 |
| 26. | CASP8 | 841 |
| 27. | CD44 | 960 |
| 28. | CXCR4 | 7852 |
| 29. | CXCL12 | 6387 |
| 30. | CDK4 | 1019 |
| 31. | GADD45B | 4616 |
| 32. | UTRN | 7402 |
| 33. | IL7 | 3574 |
| 34. | CYP7A1 | 1581 |
| 35. | CYP3A4 | 1576 |
| 36. | CYP26A1 | 1592 |
| 37. | CYP27A1 | 1593 |
| 38. | CYP27B1 | 1594 |
| 39. | CREB1 | 1385 |
| 40. | CD9 | 928 |
| 41. | NES | 10763 |
| 42. | DMD | 1756 |
| 43. | TGFA | 7039 |
| 44. | PLG | 5340 |
| 45. | HDAC1 | 3065 |
| 46. | HDAC2 | 3066 |
| 47. | HDAC4 | 9759 |
| 48. | HDAC8 | 55869 |
| 49. | MYC | 4609 |
| 50. | ASCL1 | 429 |
| 51. | IGF2 | 3481 |
| 52. | MIR9-1 | 407046 |
| 53. | LAMB2 | 3913 |
| 54. | HIF1A | 3091 |
| 55. | NREP | 9315 |
| 56. | HGF | 3082 |
| 57. | PAX2 | 5076 |
| 58. | PAX7 | 5081 |
| 59. | VIM | 7431 |
| 60. | SPARC | 6678 |
| 61. | DCN | 1634 |
| 62. | SRC | 6714 |
| 63. | PAX3 | 5077 |
| 64. | ITGAV | 3685 |
| 65. | RPS6KB1 | 6198 |
| 66. | CASP3 | 836 |
| 67. | CASP8 | 841 |
| 68. | CD44 | 960 |
| 69. | CXCR4 | 7852 |
| 70. | CXCL12 | 6387 |
| 71. | CDK4 | 1019 |
| 72. | GADD45B | 4616 |
| 73. | UTRN | 7402 |
| 74. | IL7 | 3574 |
| 75. | CYP7A1 | 1581 |
| 76. | CYP3A4 | 1576 |

**Table S3**. The list of 25 genes participated in the axon regeneration from DAVID

| **No.** | **Gene Name** | **Entrez ID** |
| --- | --- | --- |
| 1 | BRAF | 673 |
| 2 | BCL2 | 596 |
| 3 | EPHA4 | 2043 |
| 4 | LRP1 | 4035 |
| 5 | APOA1 | 335 |
| 6 | APOA4 | 337 |
| 7 | APOD | 347 |
| 8 | CHL1 | 10752 |
| 9 | DHFR | 1719 |
| 10 | GAP43 | 2596 |
| 11 | IGF1R | 3480 |
| 12 | LAMB2 | 3913 |
| 13 | MMP2 | 4313 |
| 14 | MAP1B | 4131 |
| 15 | MAG | 4099 |
| 16 | NEFL | 4747 |
| 17 | NREP | 9315 |
| 18 | NDEL1 | 81565 |
| 19 | PTEN | 5728 |
| 20 | PTPRS | 5802 |
| 21 | RGMA | 56963 |
| 22 | RTN4R | 65078 |
| 23 | SPP1 | 6696 |
| 24 | TNC | 3371 |
| 25 | TSPO | 706 |

**Table S4**. The list of 26 genes participated in the oligodendrocyte differentiation from DAVID

| **No.** | **Gene Name** | **Entrez ID** |
| --- | --- | --- |
| 1 | CXCR4 | 7852 |
| 2 | CD9 | 928 |
| 3 | SOX11 | 6664 |
| 4 | ASCL1 | 429 |
| 5 | B4GALT5 | 9334 |
| 6 | CTNNB1 | 1499 |
| 7 | CLU | 1191 |
| 8 | CNTN1 | 1272 |
| 9 | ERBB2 | 2064 |
| 10 | HDAC1 | 3065 |
| 11 | HDAC2 | 3066 |
| 12 | MTOR | 2475 |
| 13 | MDK | 4192 |
| 14 | MAG | 4099 |
| 15 | NRG1 | 3084 |
| 16 | NTRK2 | 4915 |
| 17 | NOTCH1 | 4851 |
| 18 | OLIG2 | 10215 |
| 19 | PAX6 | 5080 |
| 20 | PPARG | 5468 |
| 21 | PTEN | 5728 |
| 22 | PTPRZ1 | 5803 |
| 23 | SHH | 6469 |
| 24 | TLR2 | 7097 |
| 25 | TGFB1 | 7040 |
| 26 | TRPC4 | 7223 |

**Table S5**. The list of genes network participated in the oligodendrocyte differentiation from Cytoscape V. 3.7.0

| **No.** | Gene Name | Betweenness Centrality | Closeness Centrality | Degree |
| --- | --- | --- | --- | --- |
| 1 | CTNNB1 | 0.25045 | 0.851852 | 19 |
| 2 | NOTCH1 | 0.137452 | 0.741935 | 15 |
| 3 | PTEN | 0.041564 | 0.69697 | 13 |
| 4 | ERBB2 | 0.041757 | 0.676471 | 12 |
| 5 | PPARG | 0.031899 | 0.657143 | 12 |
| 6 | MTOR | 0.024026 | 0.657143 | 11 |
| 7 | CXCR4 | 0.026601 | 0.621622 | 10 |
| 8 | TGFB1 | 0.093136 | 0.621622 | 10 |
| 9 | SHH | 0.014978 | 0.621622 | 9 |
| 10 | OLIG2 | 0.047614 | 0.605263 | 9 |
| 11 | ASCL1 | 0.0167 | 0.589744 | 8 |
| 12 | NTRK2 | 0.031148 | 0.560976 | 7 |
| 13 | MDK | 0.013636 | 0.575 | 7 |
| 14 | PAX6 | 0.007613 | 0.575 | 7 |
| 15 | HDAC1 | 0.001581 | 0.560976 | 7 |
| 16 | TLR2 | 0 | 0.522727 | 5 |
| 17 | HDAC2 | 0 | 0.534884 | 5 |
| 18 | NRG1 | 0.010856 | 0.522727 | 5 |
| 19 | SOX11 | 0 | 0.522727 | 4 |
| 20 | PTPRZ1 | 0.023909 | 0.534884 | 4 |
| 21 | MAG | 0.00919 | 0.45098 | 4 |
| 22 | CD9 | 0 | 0.479167 | 2 |
| 23 | CNTN1 | 0.001976 | 0.469388 | 2 |
| 24 | CLU | 0 | 0.389831 | 1 |

**Table S6**. The list of genes network participated in the axon regeneration from Cytoscape V. 3.7.0

| **No.** | Gene Name | Betweenness Centrality | Closeness Centrality | Degree |
| --- | --- | --- | --- | --- |
| 1 | MAG | 0.485867 | 0.475 | 7 |
| 2 | LRP1 | 0.519981 | 0.475 | 5 |
| 3 | GAP43 | 0.054094 | 0.38 | 5 |
| 4 | APOA1 | 0.081871 | 0.38 | 4 |
| 5 | CHL1 | 0.12963 | 0.365385 | 4 |
| 6 | PTEN | 0.292398 | 0.327586 | 4 |
| 7 | NEFL | 0.105263 | 0.351852 | 4 |
| 8 | MMP2 | 0.25731 | 0.404255 | 4 |
| 9 | APOD | 0.028752 | 0.351852 | 3 |
| 10 | APOA4 | 0.015595 | 0.358491 | 3 |
| 11 | RTN4R | 0.105263 | 0.345455 | 3 |
| 12 | MAP1B | 0 | 0.345455 | 3 |
| 13 | IGF1R | 0.152047 | 0.395833 | 3 |
| 14 | EPHA4 | 0.105263 | 0.256757 | 2 |
| 15 | BRAF | 0 | 0.25 | 1 |
| 16 | TSPO | 0 | 0.271429 | 1 |
| 17 | RGMA | 0 | 0.206522 | 1 |
| 18 | SPP1 | 0 | 0.292308 | 1 |
| 19 | NDEL1 | 0 | 0.263889 | 1 |
| 20 | PTPRS | 0 | 0.260274 | 1 |


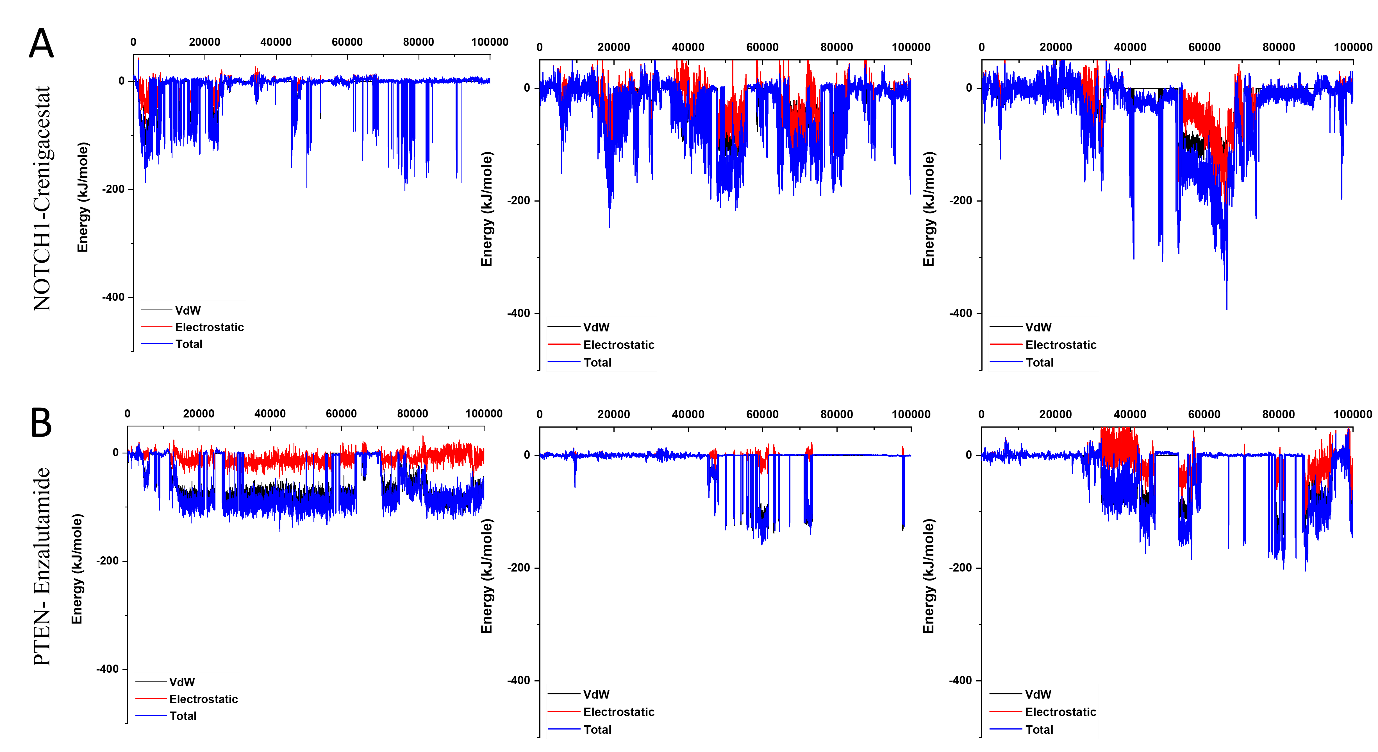


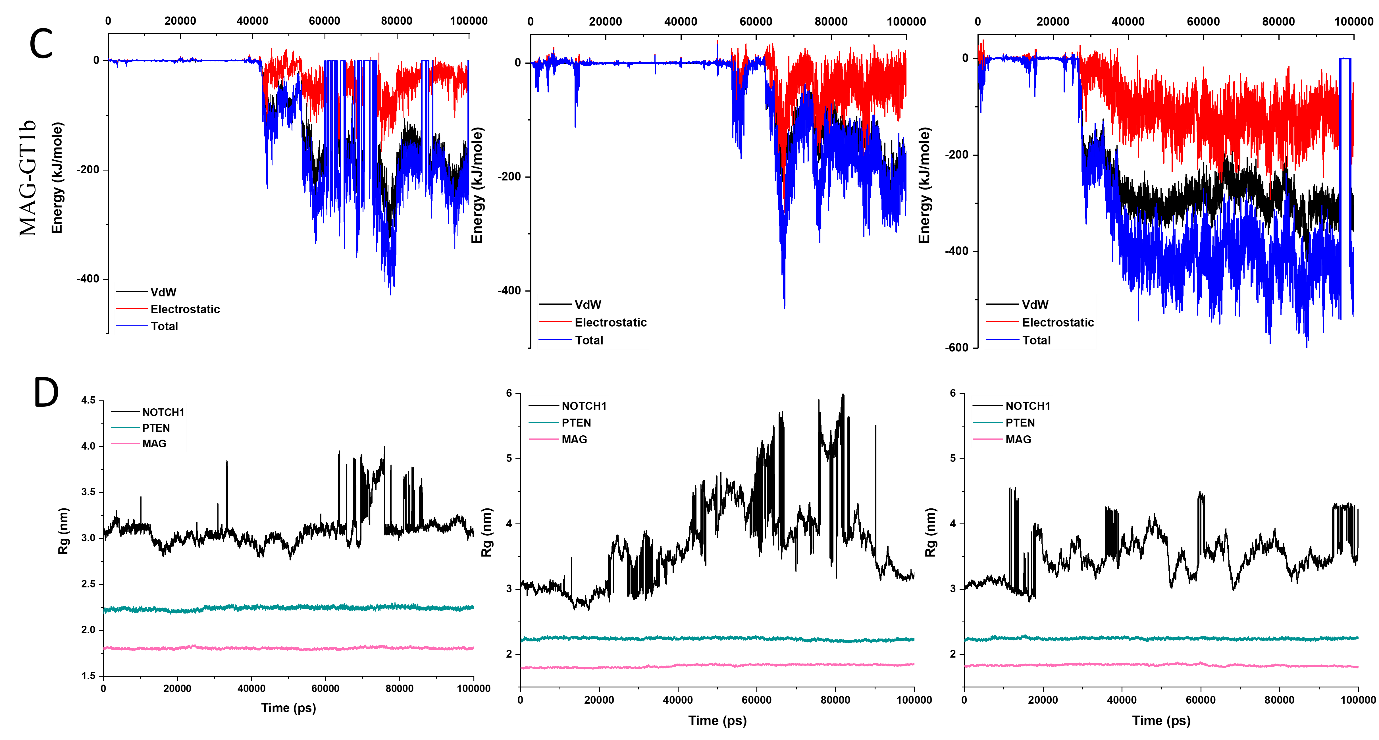


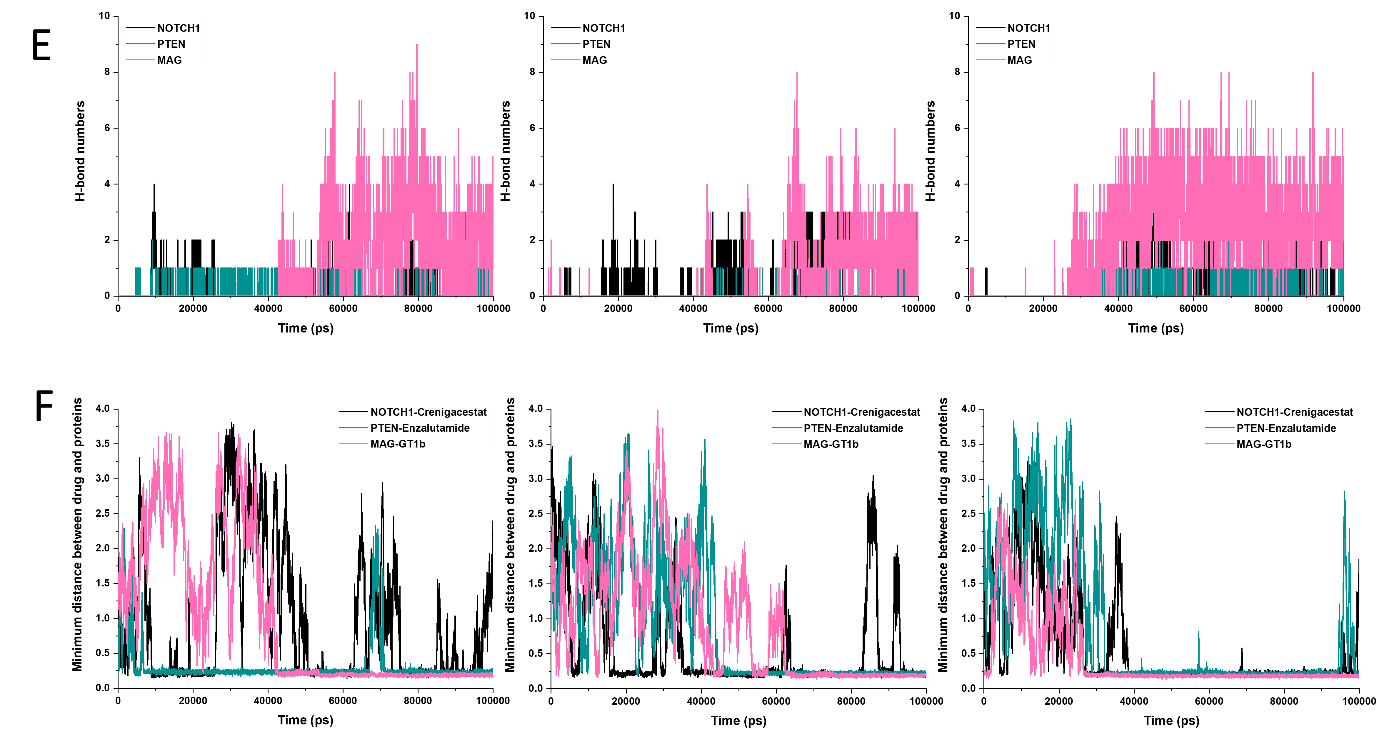


**Figure S1.** (**A-C**) The interactions energy between proteins and corresponding drugs during simulations (n=3); (**D**) Proteins’ radius of gyration during the simulations (n=3). (**E**) Number of H-bond between proteins and associated drugs at each time step during various three simulations (n=3). (**F**) The minimum distance between proteins and associated drugs at each time step during various three simulations (n=3).


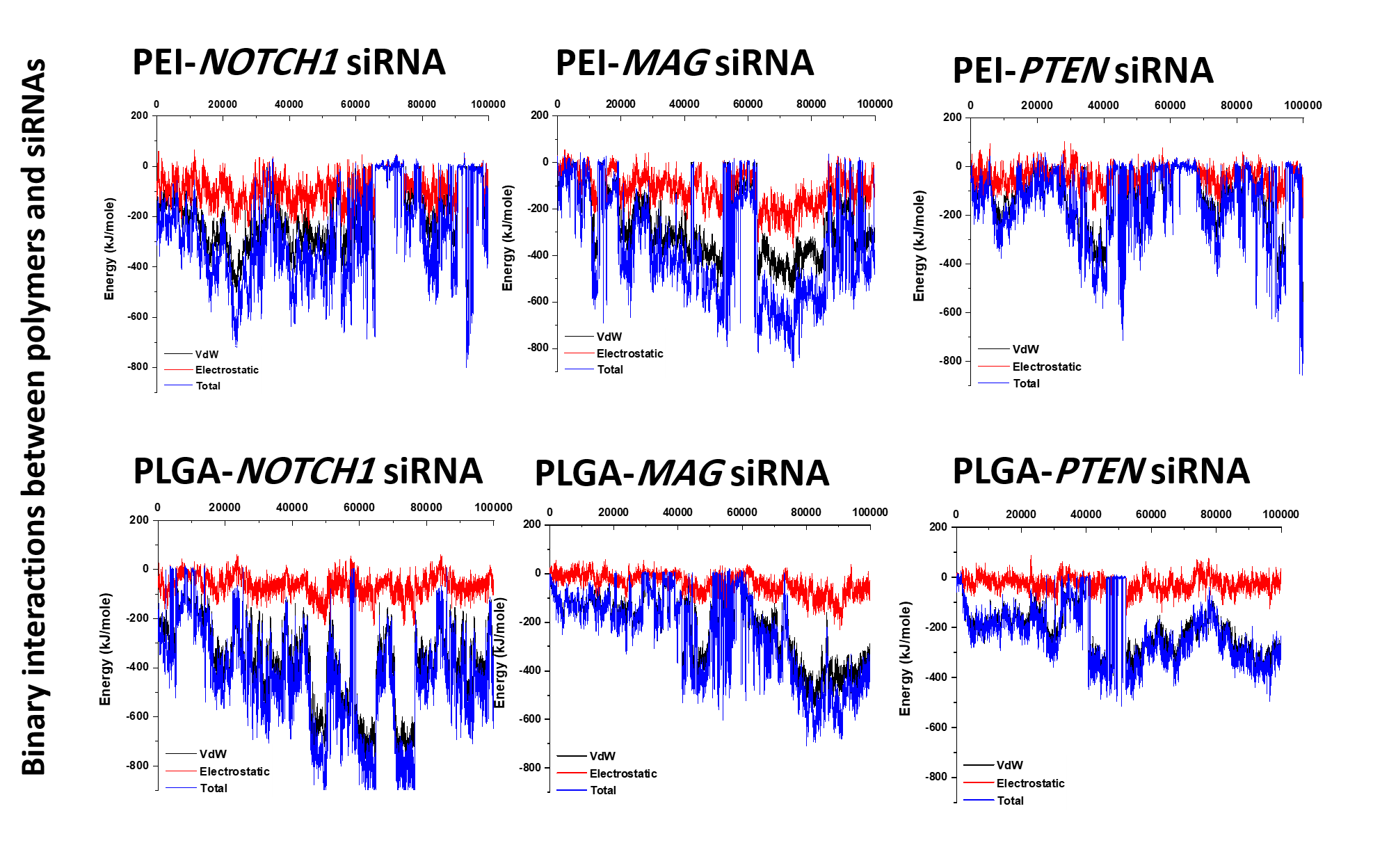


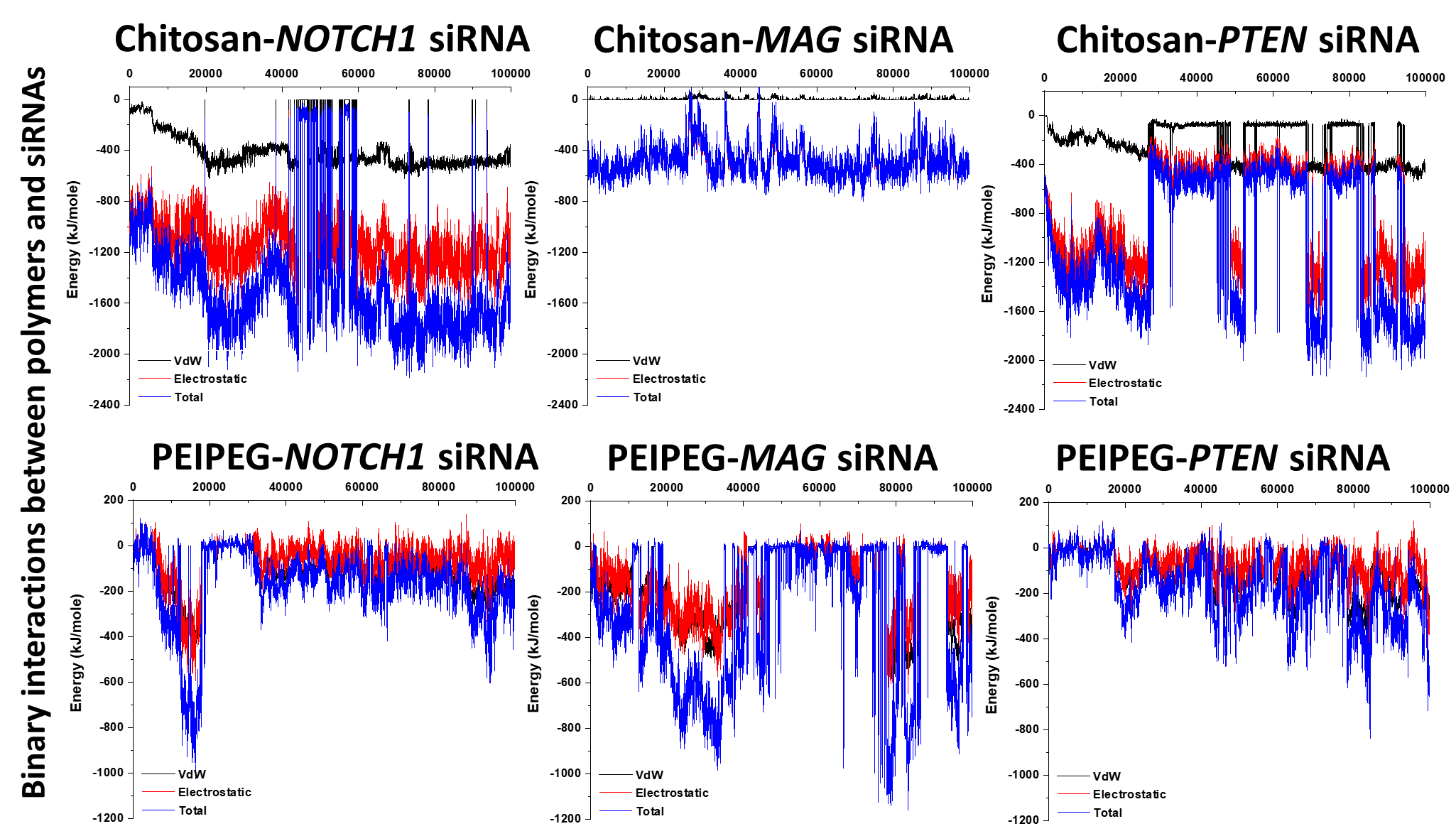


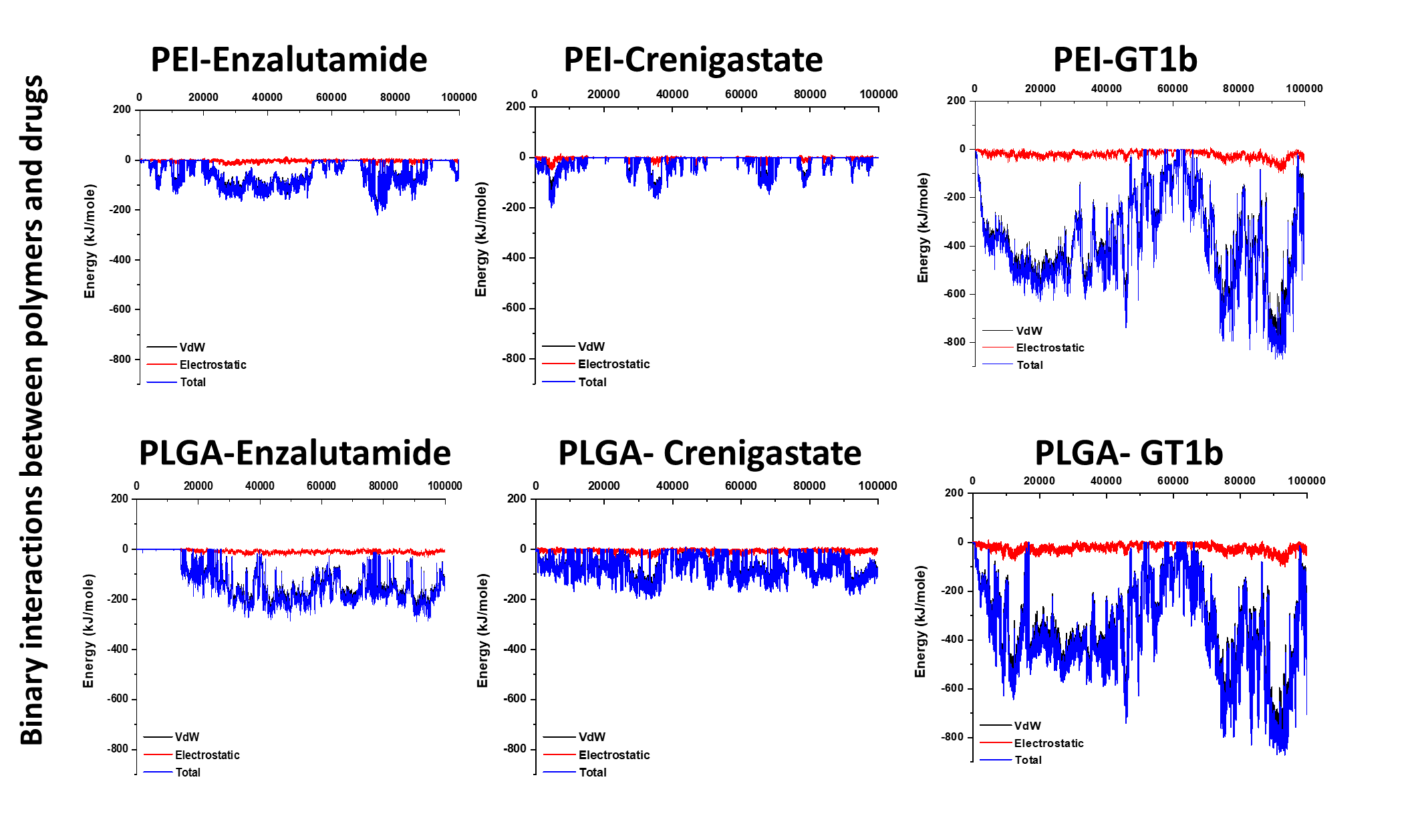


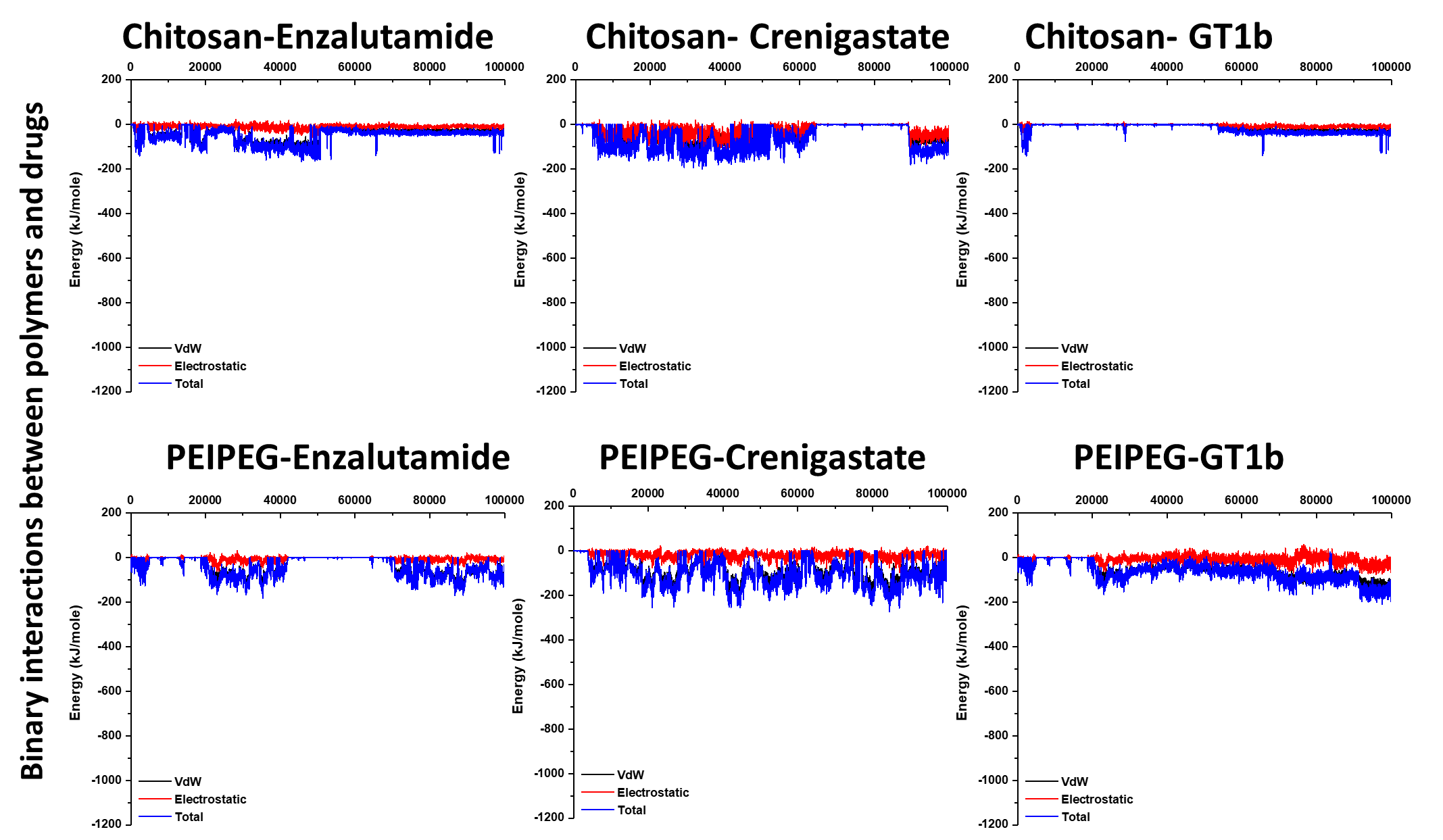


**Figure S2.** The interactions energy between polymers/siRNAs and polymers/drugs during the binary simulations.

**
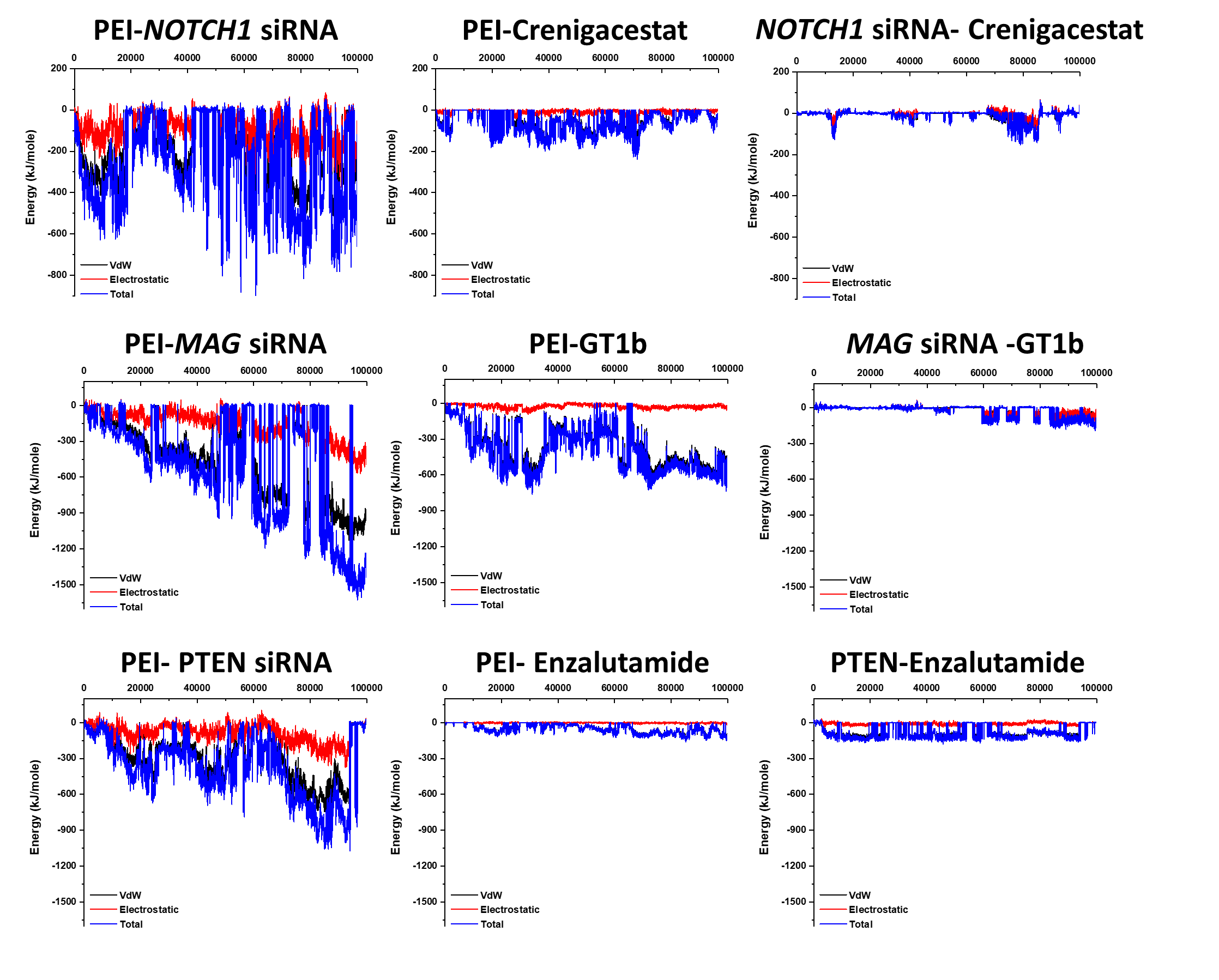
**

**
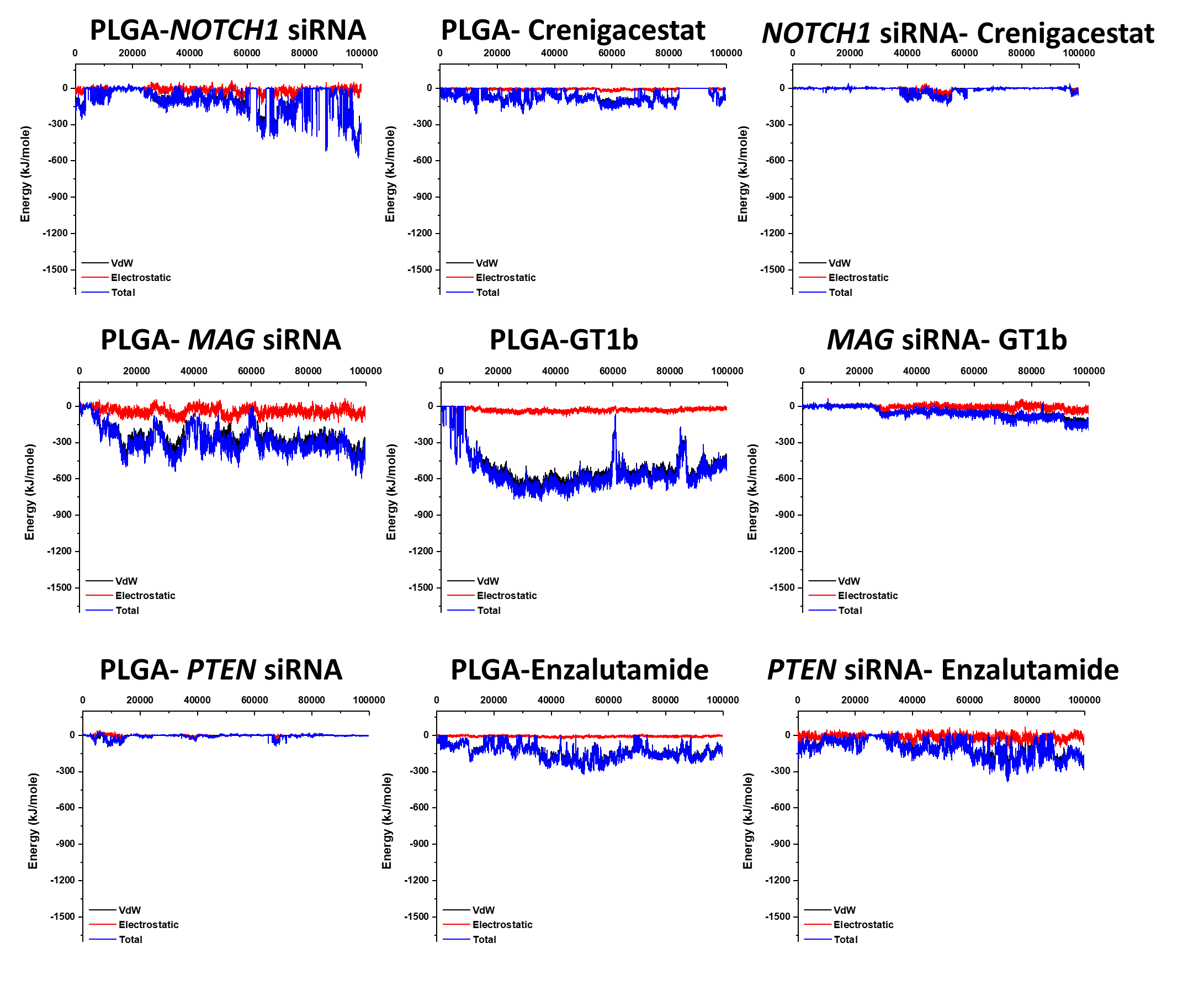
**

**
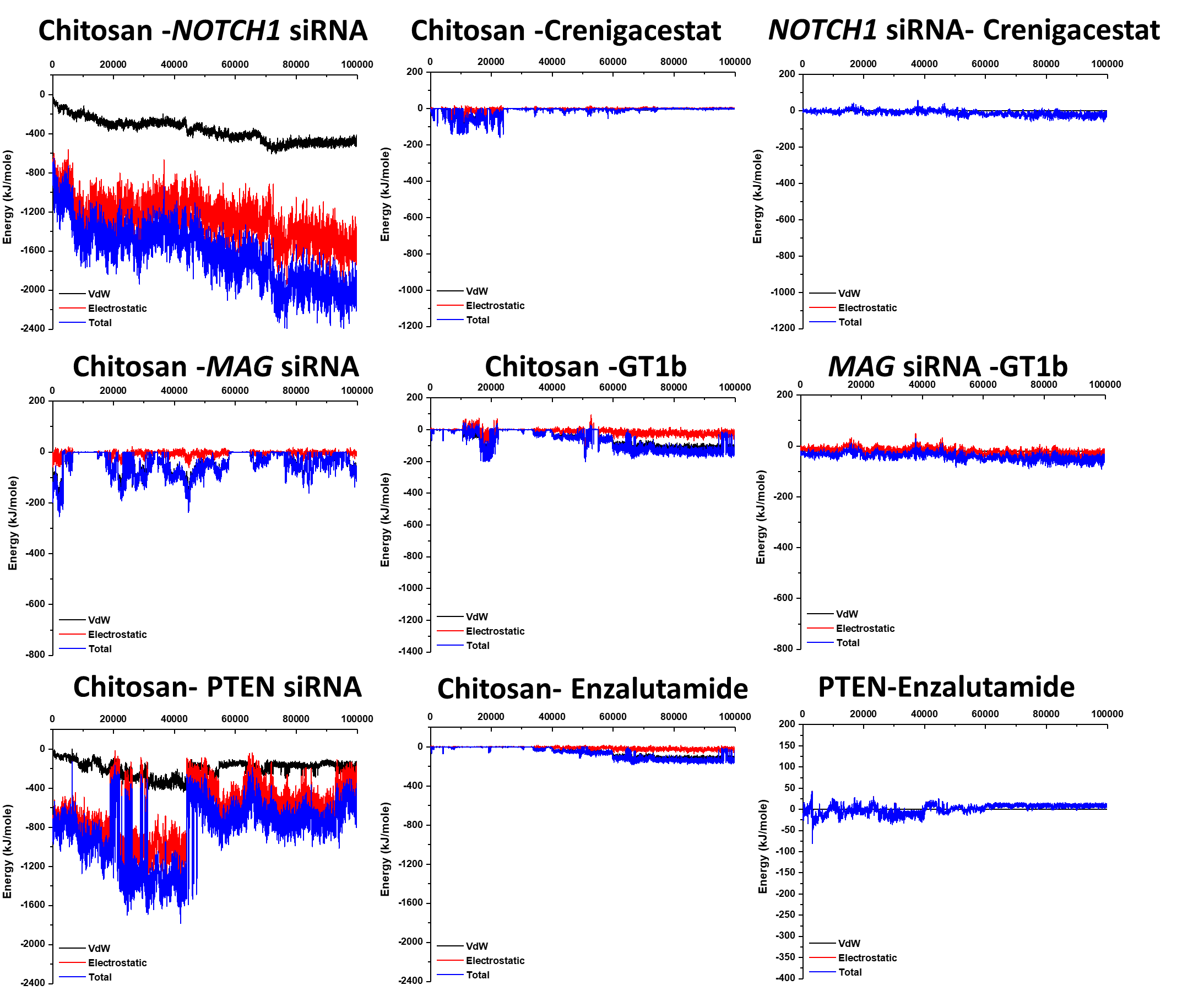
**

**
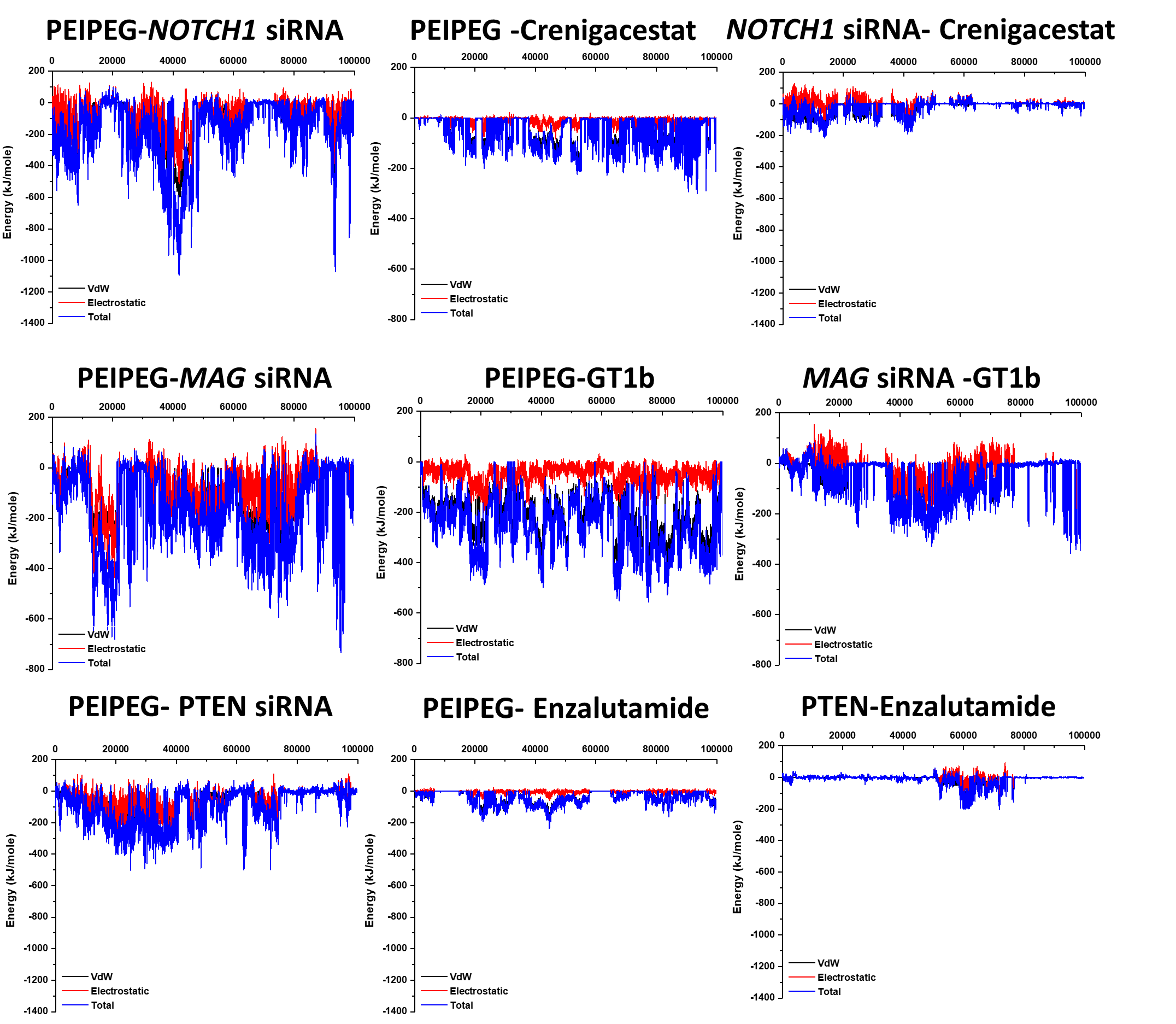
**

**Figure S3.** The interactions energy between pairs of polymers/siRNAs, polymers/drugs and drugs/siRNAs during the all-component simulations.


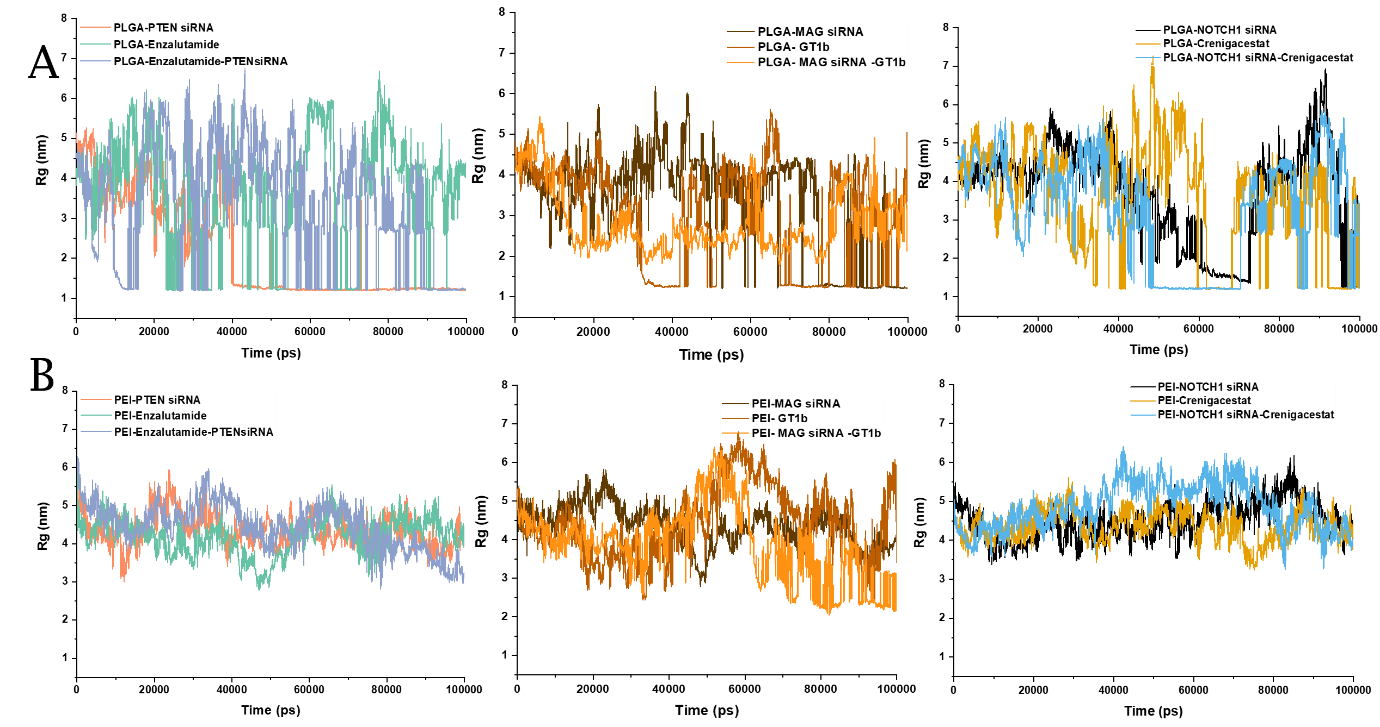


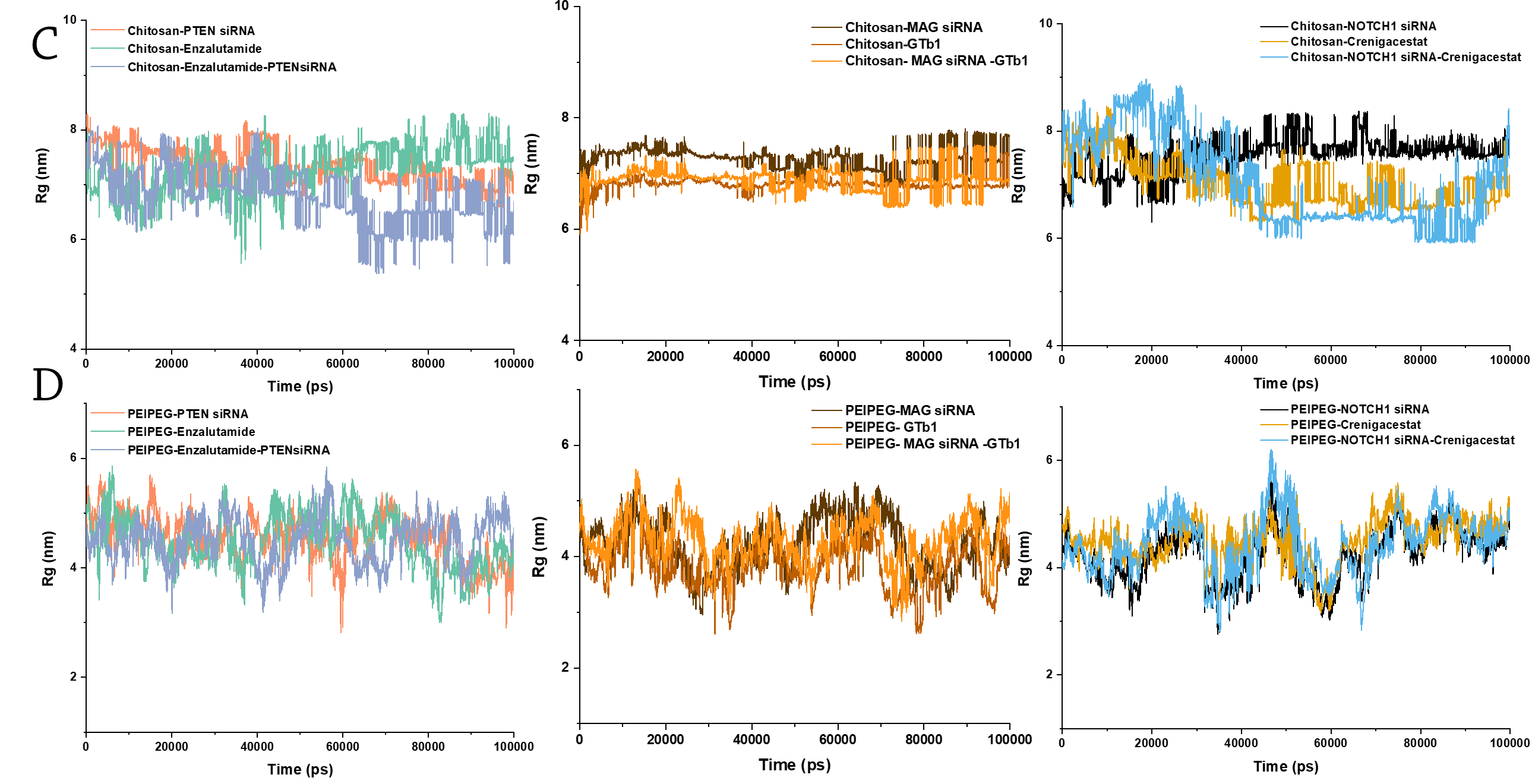


**Figure S4.** The radius of gyration during the simulation time for **(A)** PLGA-based and **(B)** PEI-based (**C**) Chitosan-based (**D**). PEI/PEG-based systems


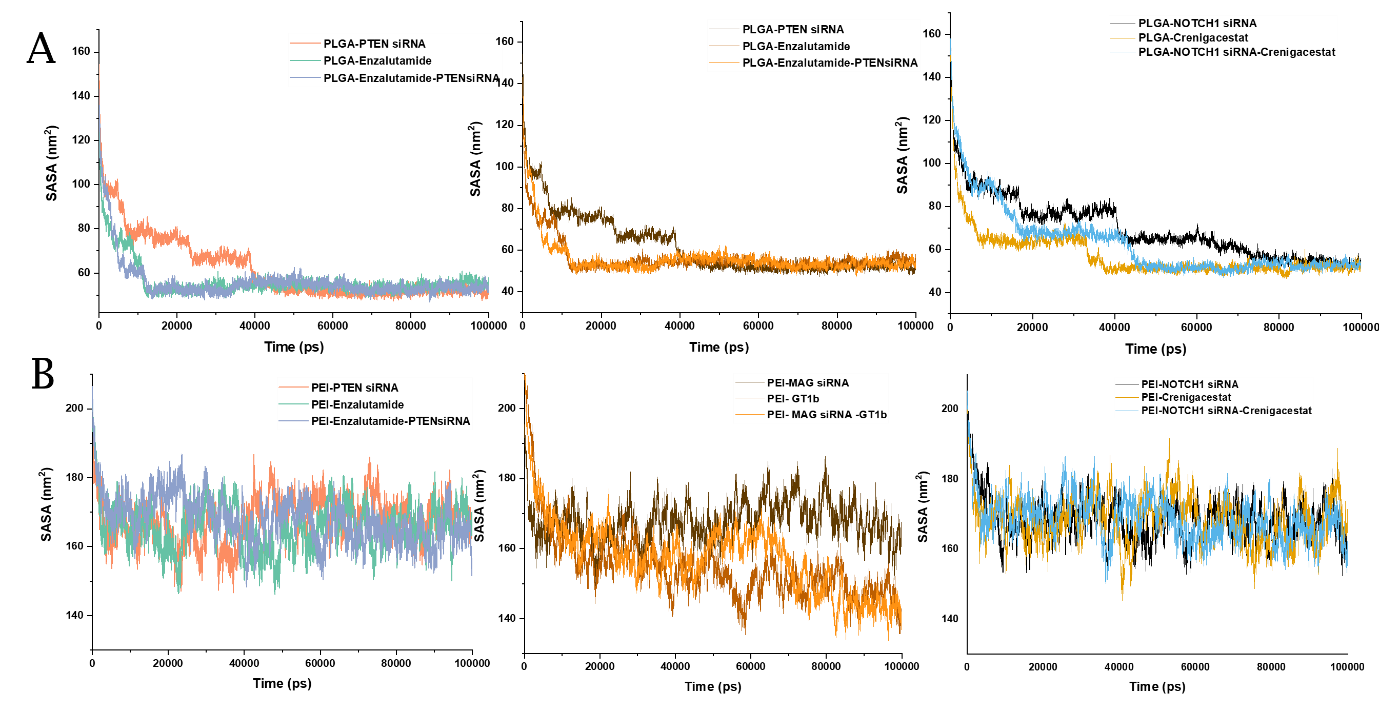


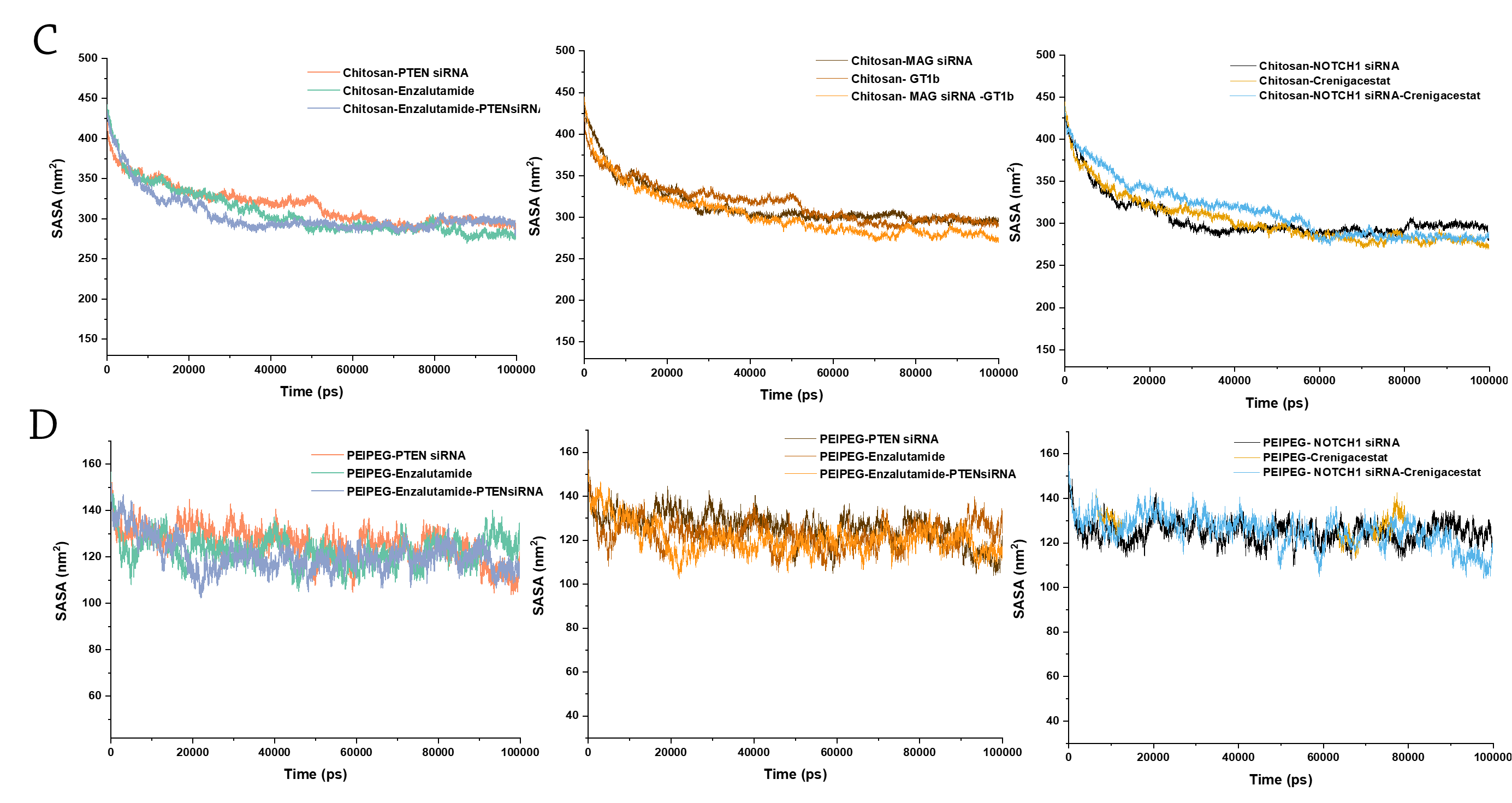


**Figure S5.** The solvent-accessible surface area during the simulation time for **(A)** PLGA-based and **(B)** PEI-based (**C**) Chitosan-based (**D**) PEI/PEG-based systems.
